# Combination Nanomedicine Strategy for Preventing High-Risk Corneal Transplantation Rejection

**DOI:** 10.1021/acsnano.4c06595

**Published:** 2024-07-29

**Authors:** Tuo Meng, Jinhua Zheng, Crystal S. Shin, Nan Gao, Divya Bande, Hadi Sudarjat, Woon Chow, Matthew Sean Halquist, Fu-Shin Yu, Ghanashyam Acharya, Qingguo Xu

**Affiliations:** † Department of Pharmaceutics, Virginia Commonwealth University, Richmond, Virginia 23298, United States; ‡ Department of Ophthalmology, Affiliated Hospital of Guizhou Medical University, Guiyang, Guizhou 550004, China; § Michale E. DeBakey Department of Surgery, Baylor College of Medicine, Houston, Texas 77030, United States; ∥ Departments of Ophthalmology, Anatomy and Cell Biology, Wayne State University School of Medicine, Detroit, Michigan 48201, United States; ⊥ Department of Materials Science and Nanoengineering, Rice University, Houston, Texas 77005, United States; # Department of Ophthalmology, Virginia Commonwealth University, Richmond, Virginia 23298, United States; ¶ Department of Pathology, Virginia Commonwealth University, Richmond, Virginia 23298, United States; ∇ Center for Pharmaceutical Engineering; Institute for Structural Biology, Drug Discovery & Development (ISB3D); and Massey Cancer Center, Virginia Commonwealth University, Richmond, Virginia 23298, United States

**Keywords:** nanoparticle, nanowafer, immunosuppressant, antiangiogenesis, ocular drug delivery

## Abstract

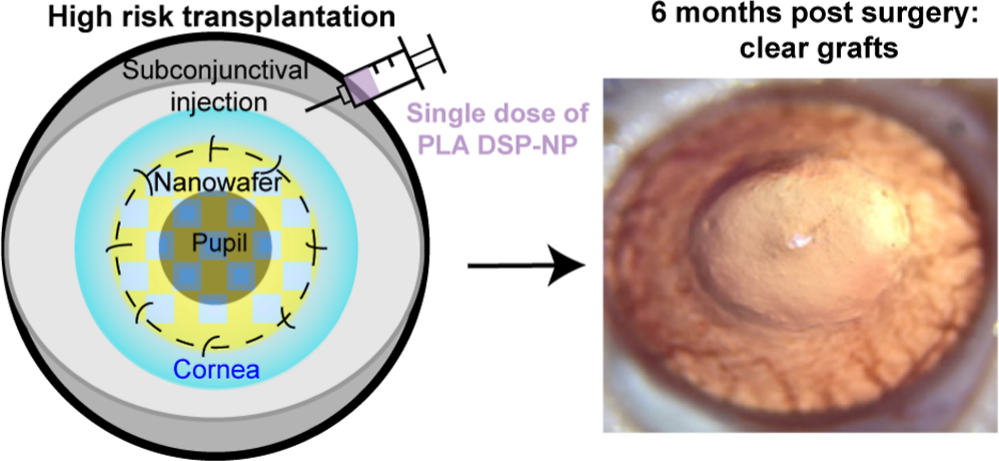

High-risk (HR) corneal transplantation presents a formidable challenge, with over 50% of grafts experiencing rejection despite intensive postoperative care involving frequent topical eyedrop administration up to every 2 h, gradually tapering over 6–12 months, and ongoing maintenance dosing. While clinical evidence underscores the potential benefits of inhibiting postoperative angiogenesis, effective antiangiogenesis therapy remains elusive in this context. Here, we engineered controlled-release nanomedicine formulations comprising immunosuppressants (nanoparticles) and antiangiogenesis drugs (nanowafer) and demonstrated that these formulations can prevent HR corneal transplantation rejection for at least 6 months in a clinically relevant rat model. Unlike untreated corneal grafts, which universally faced rejection within 2 weeks postsurgery, a single subconjunctival injection of the long-acting immunosuppressant nanoparticle alone effectively averted graft rejection for 6 months, achieving a graft survival rate of ∼70%. Notably, the combination of an immunosuppressant nanoparticle and an anti-VEGF nanowafer yielded significantly better efficacy with a graft survival rate of >85%. The significantly enhanced efficacy demonstrated that a combination nanomedicine strategy incorporating immunosuppressants and antiangiogenesis drugs can greatly enhance the ocular drug delivery and benefit the outcome of HR corneal transplantation with increased survival rate, ensuring patient compliance and mitigating dosing frequency and toxicity concerns.

Corneal transplantation is the last step in treating corneal blindness,^1^ and there are more than 180,000 corneal transplantation surgeries performed a year worldwide.^2,3^ Although corneal transplantation is the most common solid organ transplantation, the graft rejection rate can be more than 50% in vascularized and inflamed beds, called high-risk (HR) corneal transplantation.^4^ Immunological rejection is the main reason for graft failure in HR corneal transplantation,^5^ and it has been known that the preexisting blood and lymphatic vessels are the strong risk factors for subsequent immune rejection.^6^ In clinic, the HR keratoplasties are not performed in freshly vascularized and inflamed eyes but rather later in a recipient with partially regressed corneal neovascularization (NV).^44^ However, the 2 year graft rejection rate could reach 50% even with the intense topical corticosteroid therapies that require very frequent eyedrops (up to every 2 h) for at least 1 month with a gradually reduced dosing frequency over 6–12 months and a once-daily administration indefinitely.^6^ The potential explanations could be the limited pharmacodynamics of topical eyedrops (even with frequent dosing) and other substantial pathological factors, such as the feedback between angiogenesis and inflammation.

Clinical data reported that patients with HR cornea recipients induced by the corneal NV or corneal infections exhibited significantly increased levels of proinflammatory cytokines (such as IL-6, IFN-γ, and MCP-1) in both tears^7^ and aqueous humor compared with healthy volunteers or patients without pathological corneal diseases who were undergoing cataract surgery.^8,9^ It is still unclear why the corneal immune privilege is lost in HR recipients even with “partially regressed or silenced corneal NV”, while we do understand that the transient inflammatory stimulation after the HR corneal transplantation surgery can promote the progression of corneal NV by releasing angiogenic growth factors.^10,11,44^ The postoperative angiogenesis, especially the formation or reformation of lymphatic vessels, together with the recruitment of the macrophages, further enhances the alloimmune responses and promotes graft rejection in the prevascularized HR recipients.^44^ In human cornea with NV and a history of transplantation failure, lymphatic vessels with biomarkers of podoplanin and LVYE-1 have been detected, and these lymphatic vessels were associated with blood vessels and stromal inflammatory cells.^12^ As such, we hypothesized that a treatment that can effectively deliver immunosuppressants to the cornea and inhibit the corneal inflammation as well as the corneal angiogenesis post HR keratoplasty would benefit graft survival.

Tyrosine kinase inhibitors (TKI) are small molecules that target multiple angiogenesis pathways, including VEGF, PDGF, and FGF pathways, which are involved in the progress of corneal hemangiogenesis and lymphangiogenesis.^13,14^ Substantial evidence has shown the therapeutic effects of TKI in preclinical corneal NV models^15,16^ and some clinical trials are in progress (NCT05011916 and NCT01257750). The dosing regimen of TKI in clinical trials are through topical eyedrop administration with a dosing frequency of more than 3 times per day for over 3 weeks.^16^ Topical eyedrops seem to be a convenient dosage form. Nonetheless, eyedrops have limited ocular pharmacokinetic (PK) profiles due to rapid drug clearance from the corneal surface and poor corneal penetration, thus requiring multiple doses per day. The aggressive and tedious dosing regimen has been associated with poor patient adherence, and a study has shown that patient adherence to eyedrop treatment that requires more than twice dosing per day can be as low as 39%.^17^ These limitations could be even more problematic for HR recipients who already need to have intense immunosuppressant eyedrop administration.^6^ Frequent topical eyedrop administration, especially the corticosteroid, can increase the potential side effects, such as intraocular pressure (IOP) increase and cataract formation.^18^ Therefore, drug delivery systems that can achieve efficacious drug levels into the eye but with much reduced dosing frequency would improve patient compliance while satisfying the clinical outcome of HR corneal transplantation.

Here, we presented findings that the controlled delivery of an immunosuppressant and a TKI provided 6 months of efficacy in a clinically relevant rat HR corneal allograft rejection model (Figure 1). Previously, we developed dexamethasone sodium phosphate (DSP)-loaded PLA-2COOH nanoparticle (PLA DSP-NP) that provided 6 month DSP release in rat eyes following a single subconjunctival (SCT) injection.^19^ Here, we demonstrated that a single dosing of PLA DSP-NP, alone, prevented rat HR graft rejection for 6 months. We further engineered an axitinib-loaded nanowafer (Axi NW) for topical delivery of the TKI Axi. We discovered that the combination treatment of SCT PLA DSP-NP + topical Axi NW (7 dosing in total) provided significantly better efficacy than the SCT PLA DSP-NP treatment alone after HR penetrating keratoplasty (PKP) surgery. These results revealed that the potential of combination therapy strategies by targeting multiple disease pathophysiology via nanomedicine drug delivery systems enhanced ocular drug delivery to replace the need for complicated and tedious topical eyedrop dosing of corticosteroid and antiangiogenesis therapies for preventing HR graft rejection.

**Figure 1:**
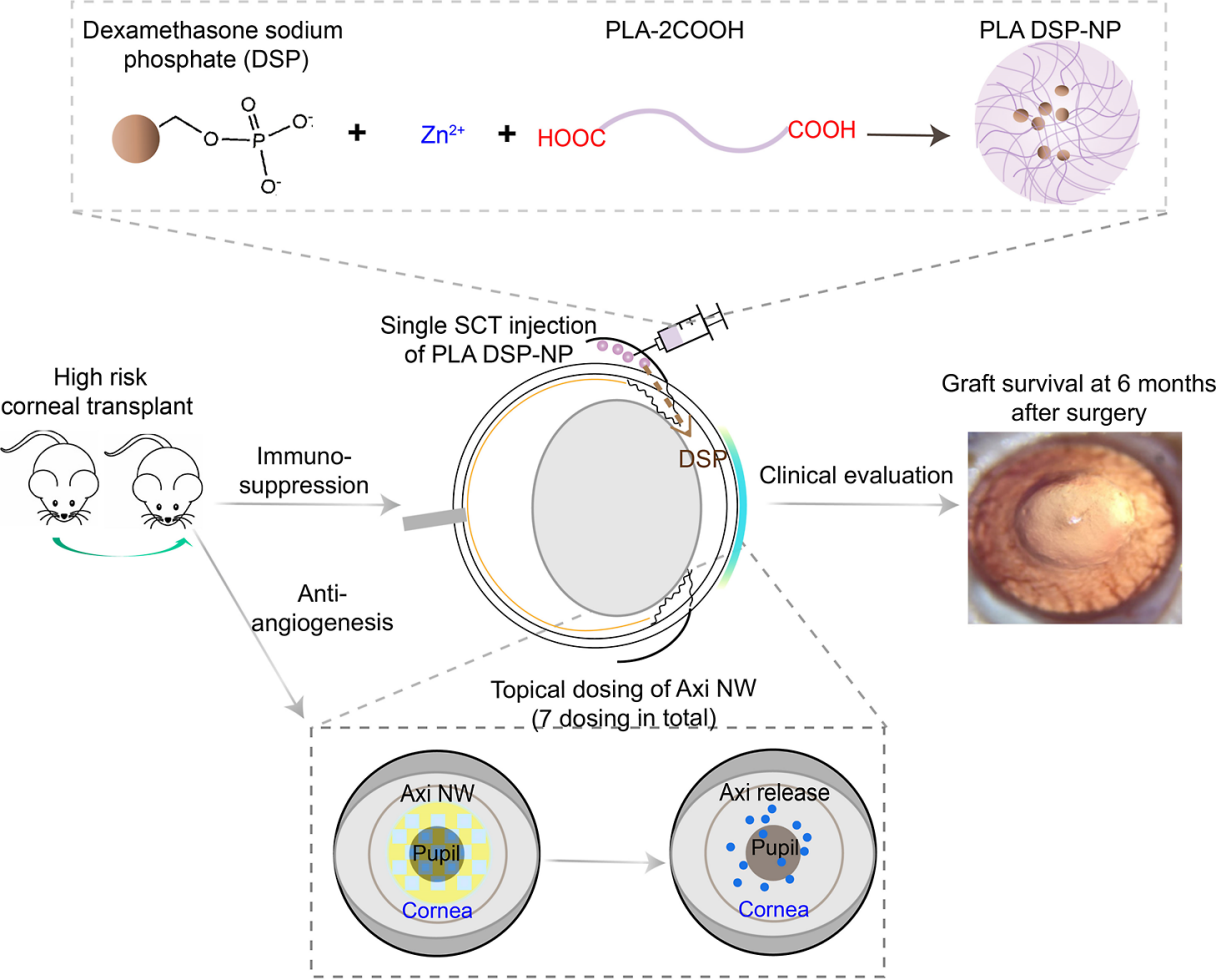
Combination treatment of long-acting PLA DSP-NP and topical Axi NW provided 6 month efficacy in preventing HR corneal transplantation rejection on rats.

## Results and Discussion

### Formulation, Characterization, and Loading Mechanism of PLA/PLGA DSP-NP

**Figure 2:**
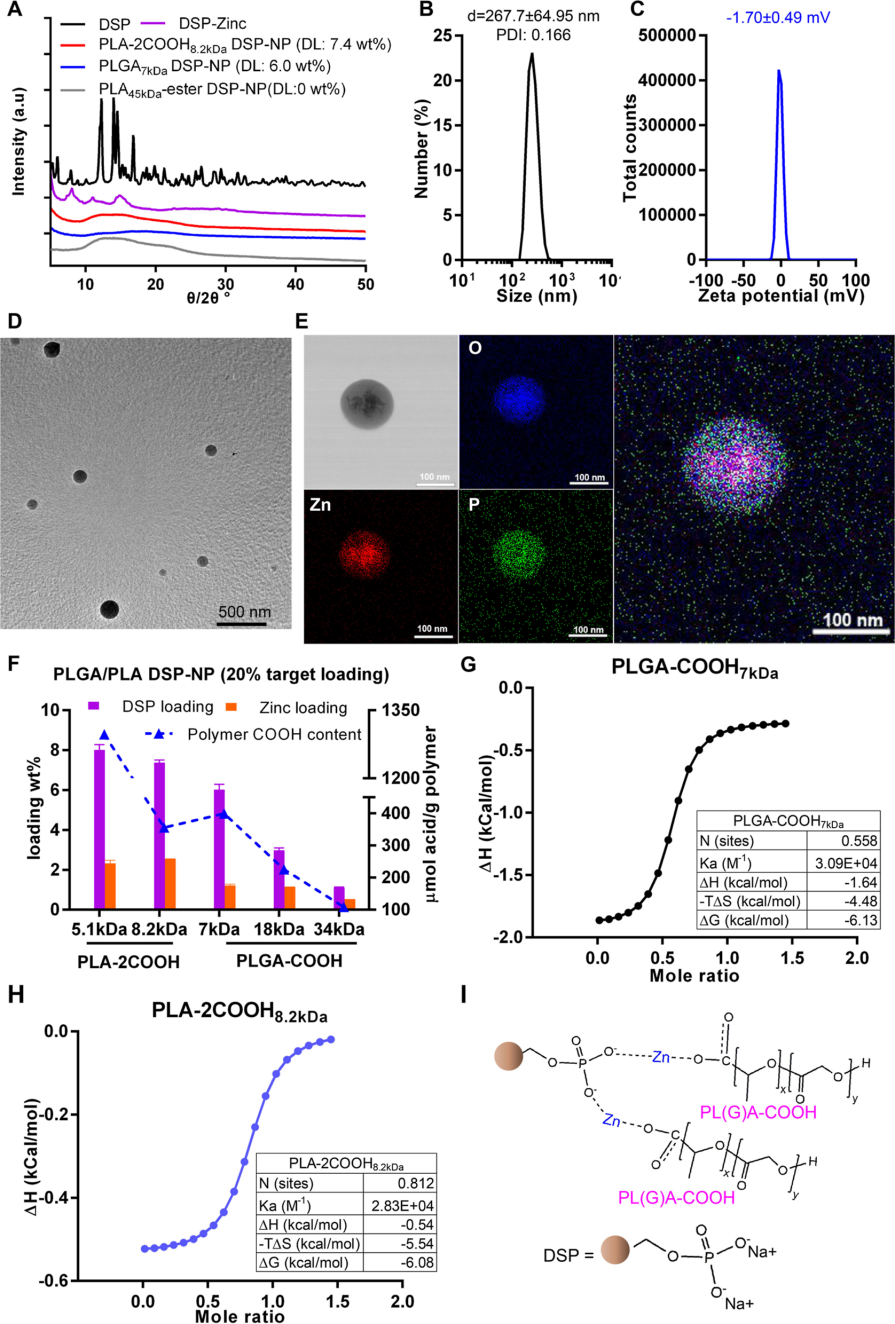
Characterization of PLGA/PLA DSP-NP. (A) XRD analysis of DSP, DSP–Zn complex, and DSP-NPs. (B,C) Particle size (B) and zeta potential (C) of PLA DSP-NP. (D) TEM image of PLA DSP-NP. (E) Elemental mapping image of PLA DSP-NP. (F) DSP and Zn loading in the DSP-NP formulated with PLGA/PLA polymers with different carboxyl contents. DSP-NP target drug loading is 20 wt % DSP. *N* = 3 for each formulation. (G,H) ITC titration of the DSP–Zn complex into PLGA-COOH_7 kDa_ (G) and PLA-2COOH_8.2 kDa_ (H) dissolved in anhydrous DMSO at 25 °C. (I) Proposed schematic image of DSP-NP.

Previously, we developed a DSP-loaded PLA-2COOH_8.2 kDa_ NP (PLA DSP-NP) that has more than 7 wt % DSP loading and sustained in vitro drug release for 3 months.^19^ To formulate PLA DSP-NP, the DSP–Zn complex was first prepared by mixing the two aqueous solutions, and the forming efficiency was more than 95% (Figure S1A). XRD analysis indicated that DSP displayed a classical crystalline structure with specific reflection in the range of 5–45°, whereas it switched to amorphous status after forming the DSP–Zn complex, indicating the coordination effects between DSP and zinc ions (Figure 2A). In the Fourier transform infrared (FTIR) spectrum, the vibrational peaks of the phosphate anion (P–O) of DSP at 1298 and 1106 cm^–1^ were shifted to 1271 and 1083 cm^–1^, respectively, after forming the DSP–Zn complex (Figure S1B). The DSP–Zn complex itself has quick dissolution under physiological conditions, leading to a rapid DSP release in vitro (>90% release within first 2 h) (Figure S1C). To achieve sustained DSP release, the DSP–Zn complex was encapsulated into dicarboxyl-terminated PLA NPs (namely as PLA DSP-NP) that have around 250 nm with more than 7 wt % of DSP loading, while no DSP was loaded into ester-terminated PLGA/PLA NPs or without the assistance of zinc ions (Figure 2A,B, Table S1). PLA DSP-NP has a nearly neutral surface charge (Figure 2C). Under 5 °C storage, there is no major change in particle size, PDI (Figure S1D) and zeta potential (Figure S1E) up to 30 days. PLA DSP-NP has a spherical shape (Figure 2D), and the DSP and zinc ions are uniformly distributed within the NPs from elemental distribution analysis (Figure 2E).

To understand the drug encapsulation mechanism of water-soluble DSP into carboxyl-terminated PLGA/PLA NPs, we formulated DSP-NP using polymers with different molecular weight (MW) and terminal functional groups. XRD analysis indicated that no crystalline form of DSP was detected regardless of the DSP loading in the NP (Figure 2A), suggesting that encapsulation of DSP is not solely dependent on the zinc-facilitated decreased crystallinity, while other factors also get involved. We found that the higher the carboxyl content, the lower the poly(D,L-lactic-*co*-glycolic acid) PLGA MW, but with the increased DSP/zinc loading in the formulated NP (Figures 2F and S2 and Table S1). Similar trends were also shown in PLA-2COOH-loaded DSP-NP (Figure 2F and Table S1). No DSP/Zn was loaded in the NPs formulated with the more hydrophobic ester-terminated PLGA polymers, even though the DSP–Zn complex was preformed (Table S1). Then, Nano ITC titration affirmed the binding between PLGA-COOH_7 kDa_ and DSP–Zn with a Ka of 3.09 × 10^4^ M^–1^, though less than the coordination bonds, still stronger than the hydrogen bonding (Figure 2G).^20^ The titration has a favorable entropy changes (Δ*S* > 0) with a favorable enthalpy (ΔH < 0) resulting in overall interaction being thermodynamically favorable (Δ*G* < 0) (Figure 2G), suggesting that the interaction of DSP–Zn---COOH-PLGA_7 kDa_ has the binding of both polar interactions (favorable enthalpy) and hydrophobic interactions (favorable entropy).^20^ Similar results were also shown in the binding of DSP–Zn and PLA-2COOH_8.2 kDa_ polymer with a Ka of 2.83 × 10^4^ M^–1^ (Figure 2H). The binding association decreases as the PLGA polymer MW increases (Figure S3), and PLGA-ester_34 kDa_ exhibited the lowest Ka of 2.49 × 10^2^ M^–1^, indicating the minimal binding affinity (Figure S3C). Differential scanning calorimetry (DSC) analysis showed an increased polymer Tg after loading DSP into the PLGA-COOH_7 kDa_ and PLA-2COOH_8.2 kDa_ NP (Figure S4). These results highlighted that the interaction between DSP–Zn and carboxyl groups on the polymer could be a leading factor for the effective loading of DSP into the NPs. The ITC titration of PLGA-COOH_7 kDa_ and PLA-2COOH_8.2 kDa_ has a stoichiometry (n) around 0.5 and 0.8, respectively. ICP-OES analysis showed that the mole ratio of zinc and phosphorus in different PLGA/PLA NPs was around 2.1 ± 0.4 (Table S2). Therefore, it is logical to expect that DSP–Zn has up to 2 residues that could interact with carboxyl groups from the polymers. The potential schematic structure of DSP-NP is updated in Figure 2I.

We previously have demonstrated that the PLGA_7 kDa_ DSP-NP only last 3 weeks which could be too short for clinical translation to prevent corneal graft rejection.^6,21^ Simply increasing polymer MW using PLGA_34 kDa_-COOH leads to more sustained in vitro drug release up to 3 months; however, only 1 wt % of DSP could be loaded due to the limited carboxyl content in the polymer.^21^ The PLA-2COOH_8.2 kDa_ DSP-NP could achieve more than 3 months in vitro drug release as well as maintaining the more than 7 wt % DSP loading.^19^ Furthermore, the PLA-2COOH_8.2 kDa_ NP provided sustained drug delivery in rat eyes for 6 months after a single SCT injection.^19^ Herein, we used PLA-2COOH_8.2 kDa_ DSP-NP (PLA DSP-NP) for the later efficacy studies.

### Fabrication and Characterization of Axitinib-Loaded Nanowafer

**Figure 3:**
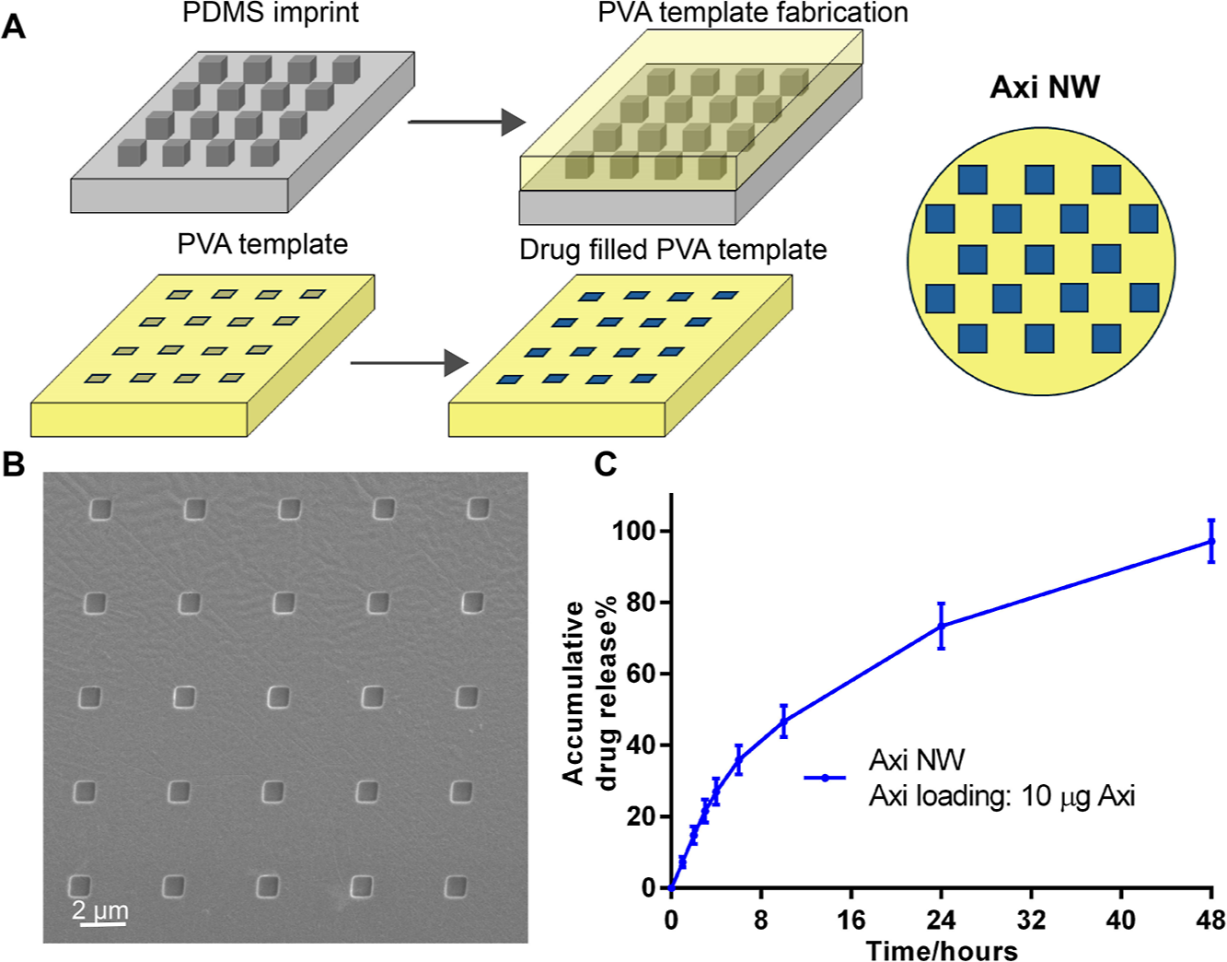
Development and characterization of Axi NW. (A) Schematic of NW preparation. (B) SEM image of Axi NW. (C) In vitro drug release profile of Axi NW. *Mean ± S*, *n* = 3 samples for in vitro drug release analysis.

Axitinib-loaded nanowafer (Axi NW) was fabricated as based on a previously described procedure with some modifications.^22^ Schematic image demonstrating the preparation of the NW is shown below (Figure 3A). PVA was selected for NW fabrication due to its water solubility, transparency, biocompatibility, and nonimmunogenicity.^22^ Axi NW has the thickness of 70–90 μm with 1 μm square wells (Figure 3B). The Axi NW has a 3.5 mm diameter to fit rat eyes. After placing on the rat’s corneal surface, the NW can be dissolved and fade away within 1 h (Figure S5A). In vitro drug release study showed that the Axi NW containing 10 μg of Axi provided sustained drug release up to 2 days with approximate 50% drug release within the first 12 h (Figure 3C).

### Ocular PK Studies of Axi-NW

**Figure 4:**
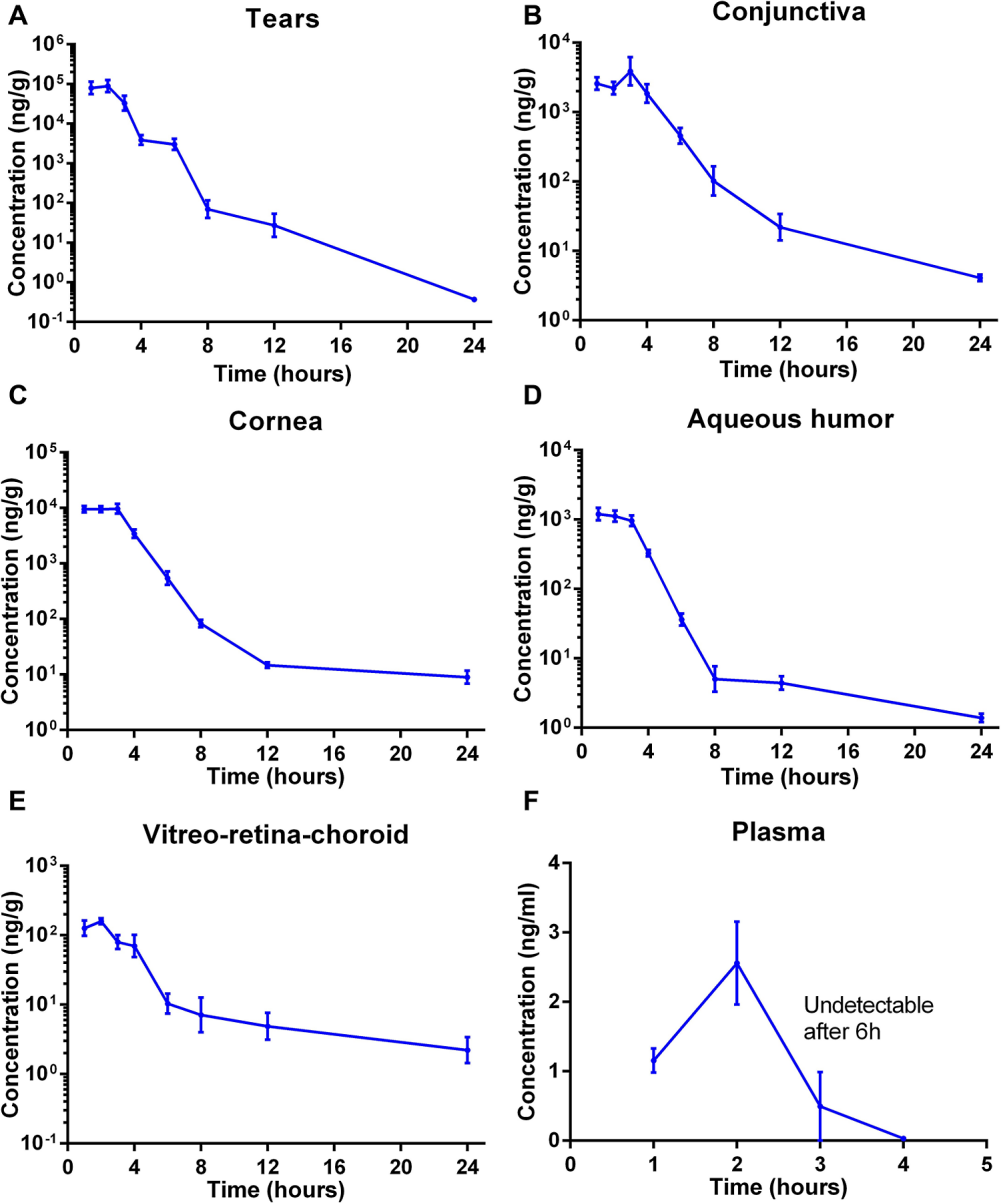
In vivo PK profiles of Axi after topical administration of Axi NW in rats. Axi concentrations in (A) tears, (B) cornea, (C) conjunctiva, (D) aqueous humor, (E) vitreo–retina, and (F) plasma at 1, 2, 3, 4, 6, 8, 12, and 24 h after topical administration of Axi NW. Mean ± SEM, *n* = 6 eyes or *n* = 3 plasma samples.

We then explored the in vivo ocular PK profiles of Axi-NW in rat eyes. Although Axi-NW was dissolved within 1 h after topical administration, it can provide sustained Axi level in tears up to 24 h after administration (Figure 4A). We observed similar trends in rat conjunctiva, cornea, and aqueous humor (Figure 4B–D). The single dosing of the Axi NW was able to provide sustained Axi delivery to the back of the eye (Figure 4E). The *C*_max_ in the all-ocular tissues was achieved at the first 3 h after Axi NW administration, and then the drug concentration was gradually diminished over 24 h. During the first 3 h, the highest Axi level was detected in rat tears followed by cornea, conjunctiva, aqueous humor, and vitreo–retina–choroid samples (Figure 4A–E). Compared to ocular tissues, only a minimal amount of Axi was detected in plasma within the first 4 h after topical administration of Axi-NW with the plasma *C*_max_ less than 3 ng/mL (Figure 4F). AUC_0–24h_ was calculated using noncompartmental analysis (Figure S5B–G). In all the ocular tissues, the highest AUC for Axi was observed in the tears, followed by cornea, conjunctiva, aqueous humor, and the vitreo–retina–choroid. AUC_0–24h_ in plasma was far lower than those in ocular compartments, highlighting the benefit of enhanced ocular drug delivery using Axi NW from both efficacy and safety perspectives.

### Construction of HR Corneal Recipient: Modified HR Recipients

**Figure 5:**
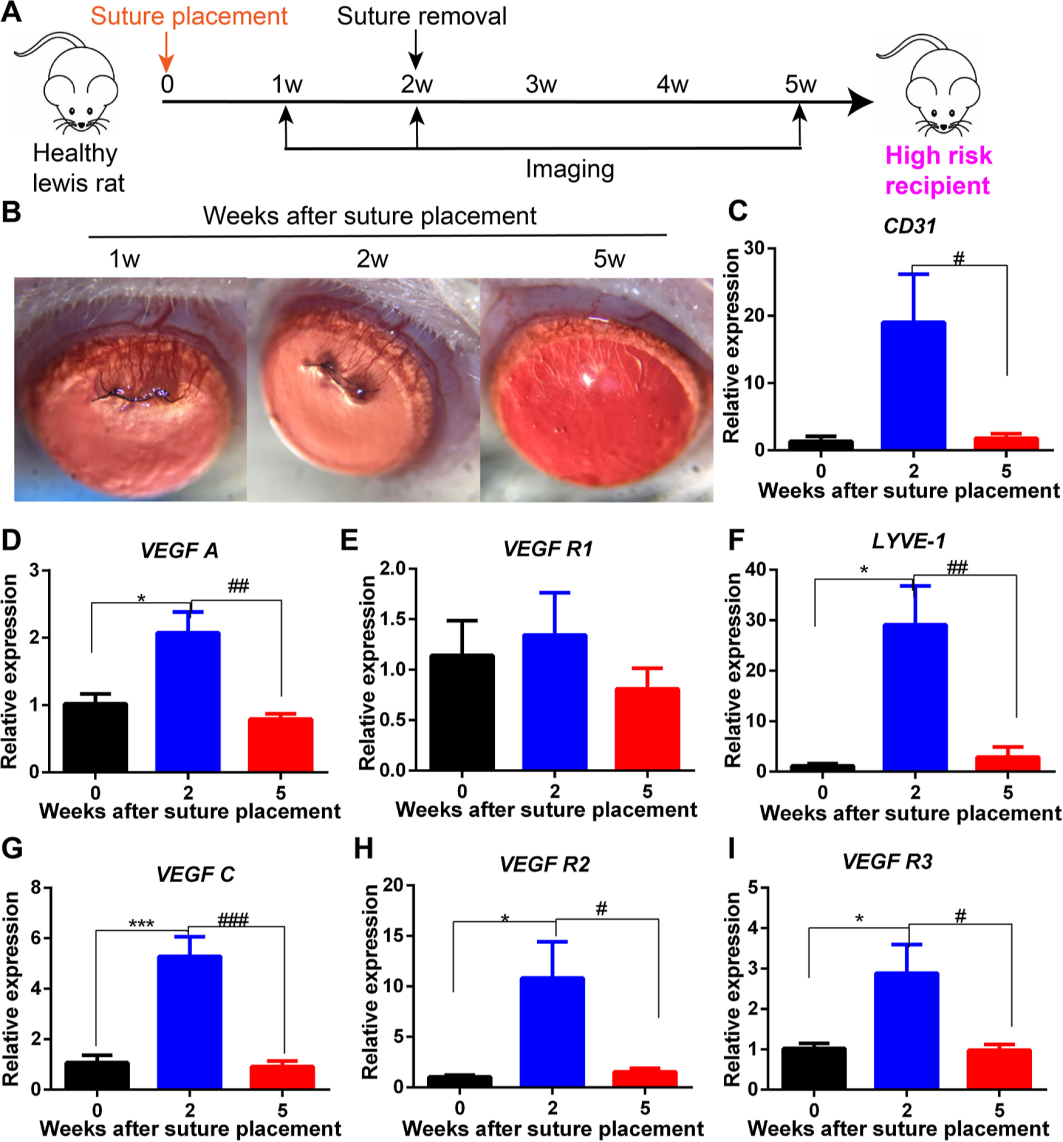
Construction and characterization of HR corneal recipients. (A) Schedule of HR-recipient construction. (B) Clinical progress of corneal NV at 1, 2, and 5 weeks after suture placement. qPCR analysis of (C) CD31, (D) VEGF A, (E) VEGF R1, (F) LYVE-1, (G) VEGF R3, (H) VEGF R2, and (I) VEGF C in the corneal samples collected at 2 and 5 weeks after suture placement. Statistical analysis for (C–I): one-way ANOVA with a Tukey post hoc test for multiple comparison. (* indicates analysis versus healthy control; *p ≤ 0.05 and ***p ≤ 0.001; # indicates cornea at 2w after suture placement versus cornea at 5w after suture placement; #*p* ≤ 0.05; ##*p* ≤ 0.01; and ###*p* ≤ 0.001). *N* = 3–6 for each group. All data are plotted from mean ± SEM.

Before we started the efficacy study, we aimed to develop a HR corneal recipient to mimic the clinical setting using the suture-induced corneal NV model (Figure 5A). After suture placed on the corneal surface, corneal NV was sprouted at 1 week and remained perfused with blood at 2 weeks (Figure 5B). Polymerase chain reaction (PCR) analysis showed that corneal hemoangiogenesis- and lymphangiogenesis-relevant genes were significantly upregulated compared with the healthy control (Figure 5C–I). Then, we removed the corneal sutures at 2 weeks, and all of the corneal NV started to regress and was silenced at 3 weeks after suture removal (Figure 5A,B). However, the ghost vessels were visible in the cornea (Figure 5B). Previously upregulated angiogenesis-relevant genes were also silenced and back to normal status at 3 weeks after suture removal (Figure 5C–I), and such a silenced cornea with ghost vessels was defined as the HR corneal recipient.

### Efficacy of PLA DSP-NP and Axi NW in Preventing HR Graft Rejection

**Figure 6:**
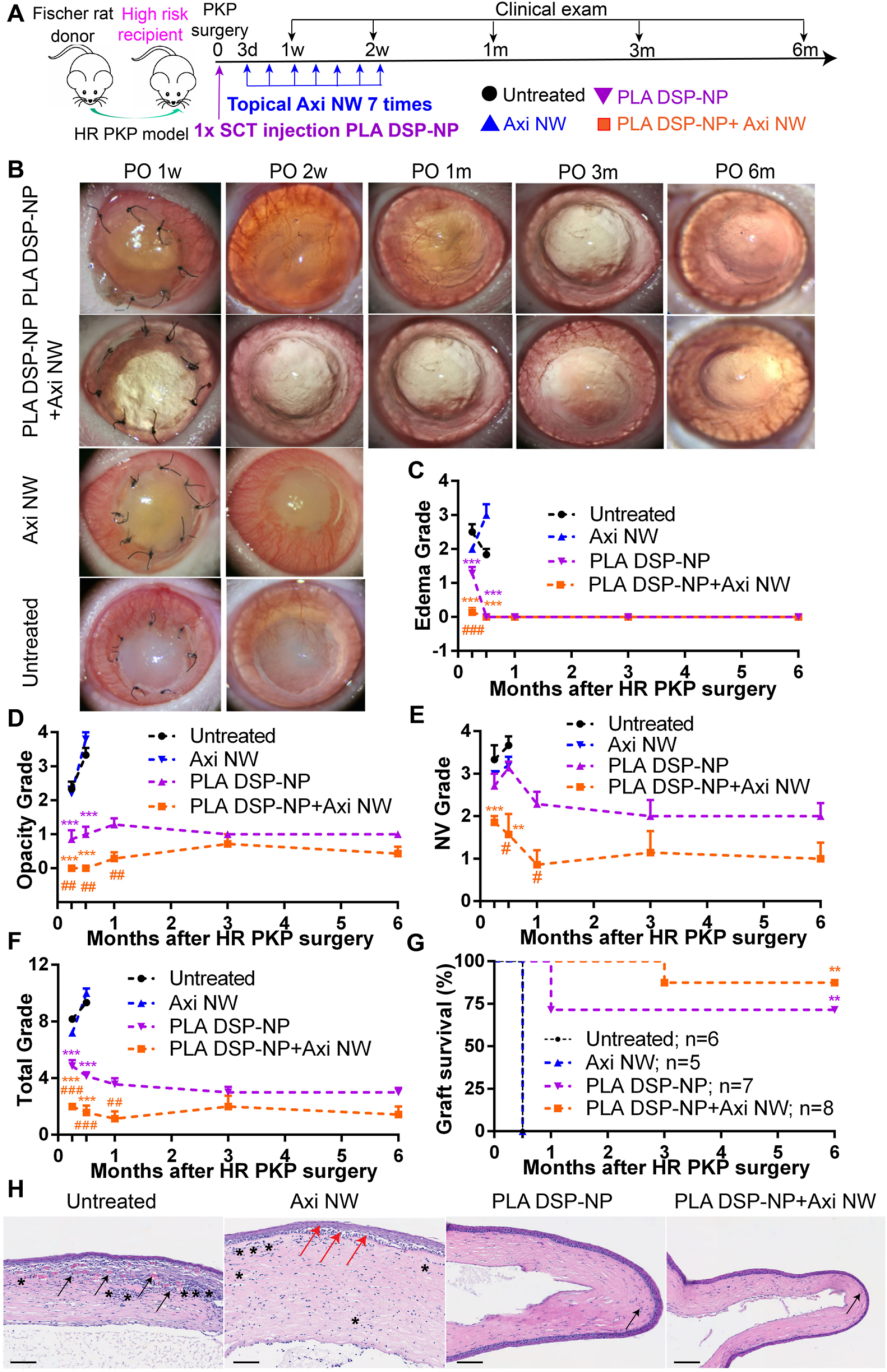
Therapeutic effect of PLA DSP-NP and Axi NW in preventing HR corneal allograft rejection. (A) Schedule for the efficacy study of PLA DSP-NP and Axi NW in preventing HR corneal allograft rejection (HR PKP surgery). (B) Representative images of grafts at 1w, 2w, 1m, 3m, and 6m after HR PKP surgery. (C) Edema, (D) opacity, (E) NV, (F) total grade, and (G) survival curves of grafts received different treatments after HR PKP surgery. (H) Histological images of grafts from different groups; scale bar 100 μm. The black arrow indicates corneal NV, the black asterisk indicates inflammatory cell infiltration, and the red arrow indicates the “bubbly” appearance of the corneal epithelial layer. Statistical analysis for (C–H): two-way ANOVA, followed by Bonferroni’s post hoc test for multiple comparison. (*p ≤ 0.05 versus untreated grafts; **p ≤ 0.01 versus untreated grafts; ***p ≤ 0.001 versus untreated grafts; #*p* ≤ 0.05 PLA DSP-NP grafts versus combination; and ##*p* ≤ 0.01 PLA DSP-NP grafts versus combination). All data are plotted from mean ± SEM.

After establishing the HR corneal recipients, we started the HR PKP surgery as previously reported.^23^ The schedule of the study is shown in Figure 6A. For PLA DSP-NP-treated grafts, a single SCT injection of PLA DSP-NP (400 μg of DSP) was administered immediately after the HR PKP surgery. Studies have shown that the initial inhibition of angiogenesis post-transplantation could prolong the graft survival.^11^ However, we concern that the exposure of Axi NW right after the surgery may impact the wound healing process of corneal grafts. Therefore, we started Axi NW treatment at postoperative day 3 (POD3) of HR PKP. Axi NWs were administered every other day for 7 times. The representative images of corneal allografts at postoperative (PO) 1 week, 2 weeks, 1 month, 3 months, and 6 months are shown in Figure 6B. At PO 1 w, untreated grafts and Axi NW-treated grafts had severe edema (average edema grade >2; Figure 6B,C) with opacity (average opacity grade >2.2; Figure 6B,D) and obvious NV invading from the bed to the graft (average grade >3; Figure 6B,E). The situation was not resolved, and all the grafts from untreated and Axi NW-treated groups were rejected at PO 2 weeks after HR PKP surgery with opacity grade more than 3 and total grade more than 6 (Figure 6B–G).

Unlike the untreated and Axi NW-treated grafts that were all rejected at PO 2w, PLA DSP-NP-treated grafts and PLA DSP-NP/Axi-NW-treated grafts were transparent during the 6 month follow-up (average grade <1.5 for both groups; Figure 6A, D). Very mild edema was only observed at PO 1w in the PLA DSP-NP only treatment group (edema grade 1.3), and no edema was found afterward (Figure 6B,C). NV was detected in both groups (average NV grade <3 for both groups; Figure 6B,E). Compared with PLA DSP-NP only treatment, the combination treatment provided significantly lower edema, opacity, NV, and total grades during the first month after the HR PKP surgery (Figure 6B–F). However, no statistical difference was detected between the two groups after PO 3m, which should be due to the termination of Axi NW treatment at day 18 after the HR PKP surgery. Only 2 grafts were rejected in the PLA DSP-NP only treatment group at PO 1m, leading to the overall survival rate of 72.5%, demonstrating the effectiveness of PLA DSP-NP in preventing HR graft rejection. However, no graft rejection scenario was found during the first month after the HR PKP surgery in the combination treatment group, and only 1 graft was rejected at PO 3 months (1 out of 8 grafts), leading to the overall graft survival rate of 87.5% during the 6 month follow-up (Figure 6G).

**Figure 7:**
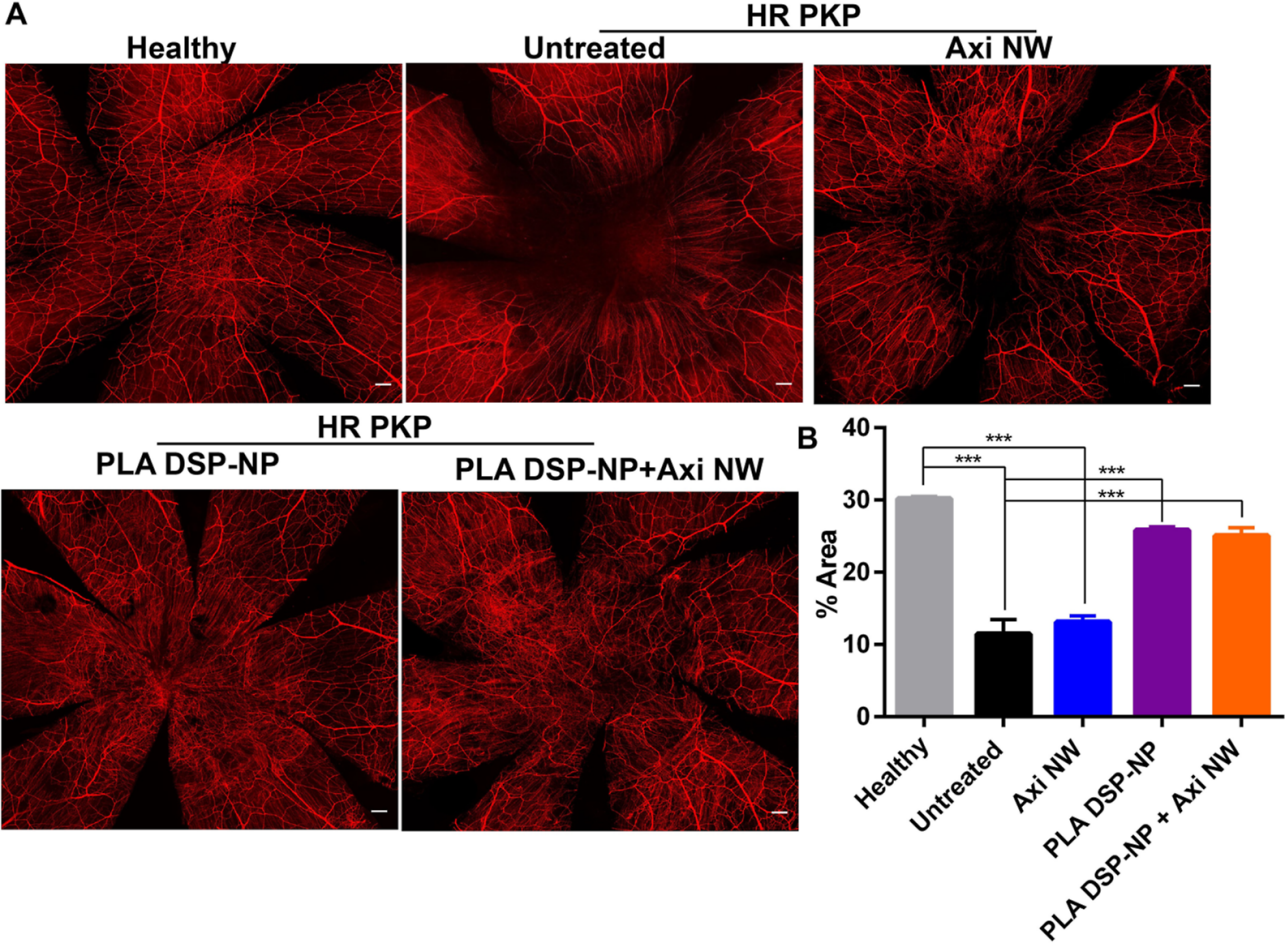
Corneal nerve regeneration after HR PKP surgery. (A) Immunostaining images of corneal nerve marker β-tubulin III, scale bar 200 μm. (B) Corneal nerve regeneration was quantified as the percentage of threshold area positive for β-tubulin III staining in the center part of cornea with the diameter of 3.5 mm. Statistical analysis for B: one-way ANOVA with a Tukey post hoc test for multiple comparison. (*p ≤ 0.05; **p ≤ 0.01; and ***p ≤ 0.001). *N* = 3 for each group. All data are plotted from mean ± SEM.

The representative histological images of grafts are shown in Figure 6H. Severe corneal inflammation (black asterisk) and notable amount of NV (black error) were shown in untreated grafts collected at 2 weeks after the surgery (Figure 6H). Similar phenomena were also observed in Axi NW-treated grafts that were collected at 2 weeks after the HR PKP surgery. The rejected grafts from both groups showed obvious edema as evidenced by the increased thickness of corneal grafts. Especially, the “bubbly” appearance of the corneal epithelia cells (red arrows) was observed in the Axi NW-treated graft, indicating the graft failure (Figure 6H). Unlike the rejected grafts, both the PLA DSP-NP and the combination-treated grafts maintained their structural integrity as evidenced by the smooth epithelium layer, intact stroma, and endothelium structure even at 6 months after the HR PKP surgery (Figure 6H). No edema and inflammatory cell infiltration were found, although a minimal amount of corneal NV was observed (Figure 6H). Corneal nerve regeneration was characterized using immunostaining of β-tubulin III. The corneal nerve in the graft was severely damaged after HR PKP surgery, and Axi NW only treatment failed to rehabilitate the nerve regeneration (Figure 7A,B). However, a notably higher degree of corneal nerve regeneration was observed in the grafts that received either PLA DSP-NP or the combination treatment (Figure 7A,B). The sub-basal nerve was regenerated in the grafts received PLA DSP-NP treatment (thin and swirl shape nerve in the center cornea), and the stromal nerve regeneration could be detected in grafts received the combination treatment (thick nerve trunks that extended and branched from the limbus to the center of the cornea).

The in vivo efficacy study indicated that the sustained delivery of DSP and Axitinib could achieve better efficacy in preventing HR corneal graft rejection post surgery but with much reduced and simplified dosing regimen than the conventional topical eyedrops. In order to confirm the combination therapy, we further studied the efficacy of PLA DSP-NP together with another TKI, suntinib malate-loaded PLGA microparticle (Sub MP), to provide sustained release for more than 2 weeks (Figure S6). In our second efficacy study, we found that the single dosing of PLA DSP-NP and Sub MP can provide 100% graft survival rate post HR PKP surgery (Figure S7). These results further support our concept that the sustained delivery of immunosuppressant and antiangiogensis therapy could benefit the outcome of HR corneal graft rejection.

## Safety Evaluation of Axi NW

**Figure 8:**
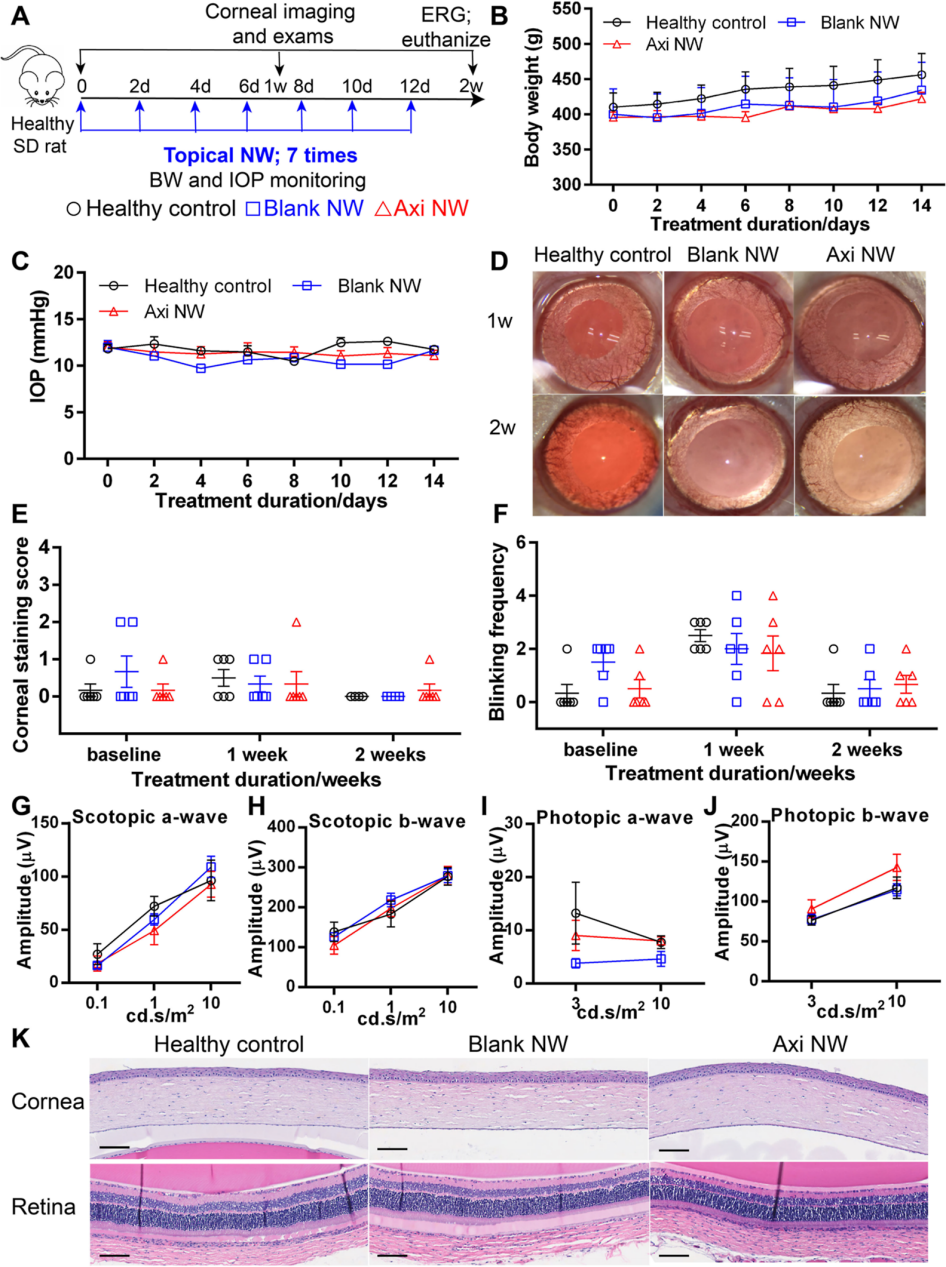
Safety evaluation of NW. (A) Schedule of safety study. (B) Corneal microscopy imaging at 1w and 2w after starting the study. (C) Rat BW and (D) IOP during the 2 week follow-up. (E) Rat cornea staining tests and (F) blink rate tests at 1 week and 2 weeks during the study. At 2 weeks after NW administration, scotopic and photopic ERG was tested. Quantification of the amplitude of (G) scotopic a-wave, (H) scotopic b-wave, (I) photopic a-wave, and (J) photopic b-wave. (K) H&E staining of cornea collected at 2 weeks after NW administration. All data are plotted from mean ± SEM (*n* = 3 for BW monitoring and *n* ≥ 4 for other tests). Statistical analysis for (B,C,E–H): Two-way ANOVA, followed by Bonferroni post hoc test.

Previously, we did comprehensive safety studies of PLA DSP-NP, and no notable systemic and ocular toxicity was found after a single SCT injection of PLA DSP-NP (400 μg of DSP).^19^ In this study, we further monitored rats’ body weight (BW) and IOP during the 6 month follow-up in efficacy studies; there was no BW reduction, and IOP increase was observed (Figure S8). Here, we mainly evaluated the toxicity of blank NW and Axi NW after repetitive topical administration (topical dosing every other day 7 times; Figure 8A). During the 2 week follow-up, no significant body weight difference was found among the 3 groups (Figure 8B). Similarly, all the rats had similar IOPs, indicating that both the blank NW and Axi NW cause no IOP increase (Figure 8C). Ocular images collected at 2 weeks after the dosing indicated that the NW did not cause cataracts after repetitive dosing (Figure 8D). No obvious difference was detected in rat corneal staining and blink rate tests among all of the groups during the 2 week follow-up (Figure 8E,F).

As the ocular PK study showed that the Axi NW delivered Axi to the back of the eye (Figure 4E), we monitored the retina function. No significant scotopic and photopic ERG differences were detected among the 3 groups at 2 weeks after repetitive NW dosing (Figure 8G–J). Histology analysis also showed similar cornea and retina structures among the 3 groups, further supporting that no obvious ocular toxicity was found after the repetitive dosing of NW (Figure 8K).

It has been known that the corneal NV is the leading factor for the graft rejection in HR corneal transplantation.^6^ The corneal bed with suture-induced corneal NV was commonly used for constructing the HR corneal graft rejection model;^24,25^ however, performing PKP surgery on vascularized corneal beds with highly perfused blood vessels is not the standard clinical practice and can have side effects of severe hemorrhage leading to postoperative complications and surgery failure. By leveraging the intermediate-risk mouse keratoplasty model established by Dr. Cursiefen’s group,^11,44^ we developed our rat HR cornea transplantation rejection model to mimic the clinical setting that the PKP surgery was performed on the noninflamed corneal bed with silenced or partially silenced corneal NV. The initiation of proinflammatory cascade postsurgery amplifies the cytokine signals and stimulates the secretion of hemangiogenic and lympgangiogenic growth factors at the injury site that allows for the rapid reconstitution of angiogenesis.^10^ The growth of lymphatic and blood vessels has been shown to recruit macrophages and contribute to the afferent and efferent arms of the immune responses after the corneal transplantation, respectively.^26^ The crosstalk between inflammation and angiogenesis can lead to the rapid donor-specific sensitization, and the lack of immune tolerogenic can cause the graft rejection within 2 weeks postsurgery.^27^ Indeed, we observed a 100% graft rejection rate within 2 weeks after the PKP surgery in our rat HR PKP model.

The clinical immunosuppressant dosing regimen for preventing HR corneal graft rejection typically includes one SCT injection of corticosteroid solution right after the surgery^28^ plus every 2 h of topical corticosteroid eyedrop during the first month with gradually reduced and eventually indefinite dosing, but such aggressive dosing regimen is still relevant to more than 50% graft rejection rate.^6^ Some clinicians prescribe systemic corticosteroid to help prevent graft rejection in HR recipients.^4^ Nonetheless, there is a lack of definite evidence to validate the effectiveness of this practice, and systemic administration of corticosteroid has been associated with unwanted systemic side effects.^29^ Topical eyedrops can only be effective when using properly; for HR transplantation, the poor ocular bioavailability of topical eyedrops and the subsequent patient nonadherence due to the tedious dosing could be one of the reasons for the high graft rejection rate.^6^ We previously developed a PLA DSP-NP using the custom-synthesized PLA-2COOH polymer and the zinc ion bridging method that provided sustained DSP release in the rat eyes for 6 months after a single SCT injection without causing the IOP increase and body weight reduction.^19^ Herein, we further demonstrated that a single SCT injection of PLA DSP-NP alone can prevent the rat HR corneal graft rejection for up to 6 months with a total dose of only 400 μg DSP, supporting the delivery of overall lower, safer but efficacious doses of DSP to the eye using PLA DSP-NP. The formation or reperfusion of the vessels postsurgery might interfere with the corneal tolerogenesis and the graft survival, and preclinical and clinical studies have shown that the early inhibition of corneal angiogenesis and inflammation can promote the graft survival rate after HR corneal transplantation.^26,30,31,44^ Bevacizumab has been used off-label for treating corneal NV as well as in the circumstance of keratoplasty.^32^ While, in a recent multicenter clinical trial, additional bevacizumab eyedrop treatment (1% eyedrop with 4 times per day dosing for 4 weeks) showed no significant improvement in 1 year endothelial graft rejection rate in HR recipients.^32^ The poor corneal penetration of a monoclonal antibody (molecular weight around 146 kDa) could be the reason for the limited efficacy of bevacizumab even with the repetitive dosing regimen.^33−35^ Compared with mAbs, TKI are small molecules that have better corneal penetration, while the topical eyedrops still required 4 times per day for more than 3 weeks (NCT05011916 and NCT01257750), which could be an “extra burden” for the HR corneal transplantation patients who already have to use every 2 h administration of topical corticosteroid eyedrops during the first month postsurgery. We previously developed the Axitinib-loaded nanowafer (Axi NW) that can be administered on the corneal surface through a fingertip to provide twice efficacy than the 2 times per day topical eyedrop dosing for treating ocular burn-induced mice corneal NV.^36^ Here, we further modified the formulation to make it applicable for rat eyes and demonstrated that the single topical administration of Axi NW provided a sustained Axi release in rat ocular compartments for 24 h with only very limited systemic exposure. The combination treatment of PLA DSP-NP and Axi NW was able to prevent HR corneal transplantation rejection for 6 months with a graft survival rate of 87.5%. Compared with PLA DSP-NP only treatment, we noticed a significant improvement in the clinical scores during the first month after HR PKP surgery. Similar results were also shown in the groups receiving the combination treatment of PLA DSP-NP and Sub MP, which further validated our concept that the effective delivery of antiangiogenesis therapies and immunosuppressants using advanced ocular drug delivery systems can improve the graft survival after HR corneal transplantation.

Unexpectedly, we discovered that both the PLA DSP-NP and the combination treatment of PLA DSP-NP and Axi NW promoted corneal nerve regeneration at 6 months after the HR PKP surgery. Corneal nerves are critical for sensation and for regulating tear secretion and blinking reflexes. Corneal nerves also play critical roles in wound repair and the maintenance of corneal surface health.^37^ Similar to the cross-talks between corneal angiogenesis and inflammation, there is a reciprocal relationship between corneal nerves and immune cells.^37^ Studies have shown that the inflammatory responses could induce nerve damage, and the damaged nerves can further aggravate immune responses.^37,38^ The sustained delivery of efficacious doses of immunosuppressant through PLA DSP-NP that inhibit the immune responses and improve graft survival could be the leading reasons for the nerve regeneration postsurgery. Some studies reported that the anti-VEGF therapies could impair the corneal nerve regeneration,^37^ while in our work, the synergistic effects of PLA DSP-NP and Axi NW could better control the immune responses and promote the graft survival as well as corneal nerve regeneration, though no significant nerve density difference from PLA DSP-NP treatment alone. More detailed studies on the regulation pathways of PLA DSP-NP and Axi NW will be performed in future work.

It needs to mention that the high drug loading of ophthalmic long-acting injectables would benefit their clinical translation due to the limited injection space of ocular tissues. The high DSP loading in our PLA DSP-NP is mediated by the noncovalent bonding between DSP–Zn and the carboxyl group on the polymer. The formation of DSP–Zn with reduced aqueous solubility created a favorable environment for the payload encapsulation into the hydrophobic NP. Nonetheless, the non-nonvalent binding between DSP–Zn and the carboxyl group on the polymer plays a dominant role in the high DSP loading in PLGA/PLA DSP-NP as evidenced by drug loading and ITC titration results. The nonvalent interactions involve in both the hydrophobic and polar interactions, though the thermodynamic presented entropy-driven interactions (|Δ*H*| < |−*T*Δ*S*|) that are typically recognized as “hydrophobic interactions”. It needs to be noted that ligand binding involves in two steps, desolvation and association. In our system, we used dimethyl sulfoxide (DMSO) as the solvent due to the limited aqueous solubility of DSP–Zn and the polymer. The hydrogen bonding between DMSO and carboxyl groups on the polymer can limit the desolvation events and the following binding of DSP–Zn and PL(G)A-COOH,^39^ giving rise to an increased solvent entropy and limited enthalpy decrease. Changing the DMSO solvent to another organic solvent such as anhydrous ethanol or using deprotonated polymers could enhance the binding and may even lead to an enthalpy-driven result.^20^ Regardless, no binding was found between DSP–Zn and the hydrophobic ester-terminated PLGA_34 kDa_, favoring our hypothesis that the interaction of DSP–Zn and carboxyl groups on the polymer is a driving factor for the high DSP in the NPs rather than the decreased aqueous solubility of the DSP–Zn complex.

We have demonstrated that long-acting ocular drug delivery systems provided promising therapeutic efficacy for preventing HR corneal transplantation rejection with much lower doses, excellent safety profiles, and improved patient compliance. All the three formulations are made by GRAS (Generally Recognized as Safe) materials and have a long history use in ophthalmic drug products. DSP-NP and sunitinib MP are mainly composed of PLA and PLGA polymers that are degraded through hydrolysis and the cleavage of ester backbone into lactic and/or glycolic acid. Under physiological condition, lactic acid and glycolic acid can be further metabolized into carbon dioxide and water through the tricarboxylic acid cycle and eliminated from the body.^40^ The drug loading and release kinetics can be further adjusted by applying different polymers or simply changing the formulation compositions. In both PLA DSP-NP and PLGA Sub MP, relatively lower MW of PLGA/PLA polymers is able to provide 6 month efficacy that can avoid unnecessary polymer retention in vivo. Axi NW is a small transparent disc fabricated with a biocompatible, water-soluble polymer, containing nanoreservoirs that can be easily modified, suggesting its clinical translation potential and broad versatility. PVA is applied for NW fabrication due to its low immunogenic properties.^22^ PVA has been used in artificial tear products. After topical administration of NW, PVA can be dissolved and fades away through eye blinking. Moreover, the drug delivery systems can be scaled up and manufactured for GMP supplies. For example, PLA DSP-NP can be manufactured through Tee-mixing which has been widely used in mRNA lipid NP vaccine GMP manufacturing; 3D printing technology can be used for the GMP manufacturing of the Axi NW.

However, there are some potential limitations for this study. First, the established HR corneal transplantation rejection model may not be able to cover the situations of severe bacterial and fungal infections or traumatic and chemical damage-driven corneal NVs.^6^ Nonetheless, we have demonstrated that effective drug delivery using nanomedicine can be critical for preventing HR corneal transplantation rejection, and the methodology applied here can be expanded to other HR corneal transplantation rejection models and generate impact in the field. We have not got the chance to study how the combination treatment influences the hemangiogenesis and lymphangiogenesis pathway and the neuroimmune crosstalk postsurgery. Detailed mechanistic studies including understanding the synergistic effects of corticosteroids and TKI on immune response, angiogenesis, and neuroimmune crosstalks could be beneficial for developing the treatment strategies for preventing HR corneal transplantation rejection.

## Conclusions

In summary, we demonstrated that both the single dosing of PLA DSP-NP and the combination treatment of PLA DSP-NP with Axi NW successfully prevented the rat HR corneal allograft rejection for 6 months. The sustained and enhanced delivery of corticosteroid and antiangiogenesis therapies using the long-acting formulations provided promising treatment strategies for preventing HR graft rejection with improved safety profiles and increased patient compliances, while without needing the tedious dosing.

## Methods and Experimental Section

### Materials

Poly(d,l-lactic-*co*-glycolic acid) (PGLA; 50:50, single carboxyl terminated) polymers with different molecular weights were purchased from Evonik (Birmingham, AL). The dicarboxyl terminated poly(d,l-lactide acid), PLA-2COOH 5.1 kDa, and PLA-2COOH 8.2 kDa, were custom-synthesized by Polymer Source INC (Quebec, Canada). DSP salt (0215756594) was purchased from MP Biomedicals (Santa Ana, CA). Sunitinib malate and axitinib were purchased from LC Laboratories (MA, USA). Pluronic F127 (a poly(ethylene oxide)–poly(propylene oxide)–poly(ethylene oxide) triblock copolymer, or PEO–PPO–PEO), poly(vinyl alcohol) (PVA) solution (*M*_w_ = 25 kDa with 88% hydrolysis), triethanolamine (TEOA, T58300), ethylenediamine-tetraacetic acid (EDTA, 324504) solution (0.5 M), zinc acetate dihydrate (383317), and all other organic solvents were purchased from Sigma-Aldrich (St. Louis, MO).

### DSP-NP Preparation

DSP-NP was prepared by the nanoprecipitation method in the presence of F127, as previously described.^19^ The DSP–Zn complex was first prepared by adding 1 mL of 0.5 M Zn acetate to a 0.5 mL DSP aqueous solution containing 20 mg of DSP. After centrifuging at 20,000*g* for 5 min, the DSP–Zn precipitate was further dissolved in 0.5 mL of tetrahydrofuran (THF) together with 100 mg of PLGA/PLA polymers. The pH of the polymer solution was adjusted to 7–8 by TEOA. The mixture was add dropwise into 75 mL of 5% Pluronic F127 aqueous solution under stirring to form DSP-NP. After THF was completely removed by solvent evaporation, 1 mL of 0.5 M EDTA aqueous solution was added to remove the unencapsulated DSP–Zn complex. The DSP-NP was collected after centrifugation at 10,000*g*, washed by 5% F127, and resuspended in ultrapure water.

### Fabrication and Characterization of a NW

A NW was fabricated according to a previous published method.^36^ First, the arrays of wells (1 μm diameter and 1 μm depth) on a silicon wafer were fabricated using e-beam lithography, followed by preparation of a poly(dimethylsiloxane) (PDMS) imprint. Then, the PVA solution was transferred to the PDMS mold. After being heated at 60 °C for 30 min, the PVA film containing 1 μm diameter wells was peeled off. For Axi NW, Axi was first dissolved in DMSO at the concentration of 400 mg/mL, followed by mixing with 5% PVA solution at the ratio of 1:3. Then, Axi/PVA solution was filled in the wells of the PVA film. The film was cut into a 3.5 mm diameter disc, NW, for later studies. The SEM imaging of Axi NW was performed on a Hitachi high-resolution analytical FE-SEM SU-70 (Hitachi Co. Ltd., Tokyo, Japan).

To measure Axi content in the NW, a piece of NW was dissolved in 1 mL of water. An additional 2 mL of ethanol was added to precipitate the PVA polymers. After centrifugation at 12,000*g* for 10 min, the supernatant was collected and filtered through a 0.2 μm syringe filter (Thermo Fisher, USA) for high-performance liquid chromatography (HPLC) analysis. An in vitro drug release study was conducted under sink conditions. Briefly, a piece of Axi NW was placed in a dialysis bag (cut off 800 kDa) with 4 mL of 0.2% Tween-80 phosphate-buffered saline (PBS) as the drug release medium. The drug release set up was placed on a platform shaker (140 rpm) in a 37 °C incubator. At certain time intervals, the whole release medium was collected and replaced with another 4 mL of release medium. The release sample collection was carried out at 1, 2, 3, 4, 6, 10, and 24 h. The Axi concentration in the drug release samples was quantified by using HPLC/UV.

HPLC analysis of Axi was performed on a Shimadzu Prominence LC system (Kyoto, Japan) equipped with a Pursuit 5 C18 column (Varian Inc., Lake Forest, CA). Isocratic separation was conducted using mobile phases containing 60% acetonitrile and 40% 10 mM NaH_2_PO_4_ at a flow rate of 1 mL/min. The eluent was monitored by a PDA detector at 331 nm. The retention time of Axi was approximately 4 min.

### Animals

All protocols are approved by the VCU Animal Care and Use Committee. All animals were handled and treated in accordance with the Association for Research in Vision and Ophthalmology (ARVO) resolution concerning the use of animals in ophthalmological research. Male Sprague–Dawley rats (SD rats, 6–8 weeks age) were used for ocular PK and safety studies. For the HR corneal transplantation studies, Fischer rats were used as donor, and Lewis rats were used as the recipient (male, 6–8 weeks age). All rats were cared for by the VCU Animal Care and Use Committee. Animals were anesthetized before euthanasia.

### In Vivo Ocular PK Study of Axi NW

Rats were anesthetized with intramuscular injections of a mixture of ketamine (80 mg/kg) and xylazine (8 mg/kg). A piece of Axi NW was placed on the rat ocular surface, and 10 μL of BSS solution was dropped to facilitate the dissolving of the Axi NW. At 1, 2, 3, 4, 6, 8, 12, and 24 h after the wafer placement, rat reflex tears were collected by installing 10 μL of BSS in the inferior conjunctival area, followed by gentle mixing, and reflex tear fluid was collected using a 5 μL volume glass capillary tube (Drummond Scientific, Broomhall, PA, USA) by capillary action from the interior tear meniscus in the lateral canthus.^41,42^ After tear fluid collection, the rats were euthanized, and 500 μL of whole blood was collected through heart puncture and stored in BD Vacutainer blood collection tubes with EDTA coating (NJ, USA). A piece of conjunctiva tissue and the whole eyeballs were collected. Various ocular compartments including corneal, aqueous humor, and vitreous humor were obtained through eyeball dissection under frozen conditions. Plasma was collected by centrifuging whole blood samples at 1000*g* for 15 min. The biological samples were stored at −80 °C before LC/MS/MS analysis.

### HR Corneal Transplantation

The HR corneal transplantation (HR PKP) model was established according to a previous reported method with certain modifications.^43^ To construct the HR PKP model, the suture-induced corneal NV model was first developed.^15^ In brief, male Lewis rats (6–8 weeks) were anesthetized, and topical installation of 0.5% tropicamide eyedrops was used for pupil dilation. Then, three intrastromal 10-0 nylon suture (Surgical Specialties Co., Wyomissing, PA, USA) stitches were placed in the superior cornea of the eye. The distance between the stitch and the limbus–cornea boundary was approximately 2 mm, and the distance between the two stitches was 1.5 mm. At 1 week after suture placement, the corneal NV growth was significant. At 2 weeks after suture placement, rats with corneal NV were randomly distributed into different groups, and then sutures were removed.

The HR corneal allograft transplantation was conducted at 3 weeks after suture removal, confirming that the active corneal NV was silenced. The PKPs were performed by an experienced corneal surgeon under an operating ophthalmic microscope (Zeiss, Germany) as described before.^23^ In brief, Fischer donor rats were sacrificed, and the central corneal button of both eyes was removed with a 3.5 mm trephine and kept in a physiological solution ready for use. The cornea-recipient Lewis rats were anesthetized, and the pupils were dilated by 0.5% tropicamide eye drops. The paracentesis was performed before trephinization, and the anterior chamber was filled with Healon GV (Johnson & Johnson, USA). The corneal buttons were removed from the receptor Lewis rats with the 3.0 mm trephine. The donor corneal buttons were sutured to receptor corneas with eight interrupted sutures using 10-0 Nylon.

Right after the HR PKP surgery, a single SCT injection of PLA DSP-NP (400 μg of DSP) or Sub MP (150 μg of Sub) was performed. Starting at POD3, the topical administration of Axi NW was performed once every other day 7 times.

### Clinical Evaluation

Rats were immediately sacrificed when severe graft infections, bleeding, and cataracts were shown. Three parameters were evaluated for the examination of the corneal grafts including corneal transparency, edema, and neovascularization. Detailed clinical scores are listed in Table S4. Grafts were considered to have been rejected only when the total score reached 5 with an opacity score ≥ 3. The scoring system was the same as described before.^23^ Digital pictures were taken under a microscope using an iPhone 8.

### PCR Analysis

At 1, 2, and 5 weeks after suture placement, rats were sacrificed, and the cornea samples were collected for PCR analysis. Cornea was first cut into small pieces and further homogenized in 400 μL of TRIzol (Thermo Fisher Scientific, USA) using a Bullet Blender Storm (Next Advance, USA). After centrifugation, the supernatant was collected and extracted with 80 μL of chloroform, followed by further extraction using isopropanol. Finally, the RNA was precipitated and resuspended in water. After quantification, the complementary DNA (cDNA) was generated by using a high-capacity cDNA reverse transcription kit (Applied Biosystems, USA). PCR analysis was conducted in three replicates using a SYBR Green Master Mix (Applied Biosystems, USA). Primer sequences can be found in Table S3. The ΔΔCT method was used to analyze gene expression, normalized to the reference gene GAPDH.

### Histological Examination

At the end of the studies, rats were sacrificed, and the eyes were enucleated. The whole eyeballs were fixed with 10% formalin for 24 h before paraffin embedding. Axial sections (5 μm) with anteroposterior orientation (from the cornea to the optic nerve) were cut and stained with hematoxylin and eosin. The slides were analyzed and graded by an ophthalmic pathologist under masked conditions.

### Whole Mount Staining of Corneal Nerve Fibers

Rat eyeballs were collected immediately after sacrificing the rats. The detailed sample process was based on the method mentioned previously.^44^ Then, the eyeballs were fixed with 4% PFA at room temperature for 1 h. A hole was made at the retinal side, vitreous were removed, and the eyeballs were fixed again in 4% PFA for another 30 min. Cornea plus the limbus was excised under the operating microscope. Before staining, 6 radial incisions were made to allow the tissue to lay flat. The corneas were incubated at 37 °C in 20 mmol/L EDTA for 30 min and then permeabilized with the solution containing 0.025% hyaluronidase and 0.1% EDTA at pH 5.3 for 3 days. After that, cornea samples were washed with PBS with 0.3% Triton X-100 (Sigma-Aldrich) for 4 × 10 min followed by blocking with 2% BSA-PBS-Triton X-100 solution at room temperature for another 2 h. The cornea was incubated at 4 °C overnight in fluorescein-labeled antirat β-tubulin III antibody (R&D systems, Cat No: NL1195R). The following morning, the corneas were rinsed 4 times for 10 min each in PBS with 0.3% Triton X-100 and 0.05% Tween-20. Then, the tissues were cover-slipped with mounting medium (Vectashield; Vector Laboratories, Burlingame, CA) and examined under a confocal microscope (TCS SP2; Leica, Heidelberg, Germany). The density of nerve in the center of cornea was quantified using ImageJ. Basically, images were converted to black-and-white images, and the center of the cornea with a diameter of 3.5 mm was selected as area of interest, and the nerve density was measured and represented as the percentage of each center area occupied by nerves.

### Safety Study of Axi and Blank NW

Healthy SD rats were used for safety evaluation of NW. Baseline body weight, IOP, and ocular images were first collected before NW administration. Normal control rats that received no treatment were used as negative control. Rats in blank NW and Axi NW groups were given a piece of NW every other day for 14 days (7 times in total). NW was administered under anesthesia situations. Rat BW and IOP were monitored every other day. At 7 and 14 days after NW administration, microscopic images were taken, and rat blink rate and cornea staining tests were conducted. For the blink rate tests, rats were restrained gently, and the blink rate was determined by counting the number of blinks within 3 min. A drop of lissamine green (1% BSS solution) was applied to the ocular surface, and the cornea staining was scored as previously reported by the clinician.^45^ Retinal function was assessed using ERG analysis. At the end of the studies, rats were sacrificed, and the eyes were collected for histological analysis.

### Statistical Analysis

Statistical analysis for clinical score evaluation was performed by either one-way analysis of variance (ANOVA) followed by Tukey’s test under multiple comparison (α = 0.05) or two-way ANOVA analysis followed by Bonferroni multiple comparison (IBM SPSS version 26, NY, USA). The Kaplan–Meier method was used to assess the graft survival rates. All figures were prepared using GraphPad Prism, and data were displayed as the mean ± standard error of the mean (SEM).

## Data Availability

All data needed to evaluate the conclusion are presented in the paper and/or the Supporting Information. The data that support the findings of this study are available from the corresponding author upon reasonable request.

## Supporting Information Available

DSP-NP characterization, powder X-ray diffraction, FTIR, DSC, ITC, elemental quantification, carboxyl content determination, Axi-NM dissolution, formulation of Sub MP, IOP, pharmacokinetics of Axi, and efficacy of DSP-NP and Sub MP (PDF)
