# Supplementary material for: Combination Nanomedicine Strategy for Preventing High-Risk Corneal Transplantation Rejection
Source: ACS Nano. 2024 Jul 29;18(31):20679–93. doi: 10.1021/acsnano.4c06595 (PMC11308920; doi:10.1021/acsnano.4c06595)
Supplement: Supplementary file 1 — nn4c06595_si_001.pdf [file nn4c06595_si_001.pdf]

Supplementary Materials

Combination nanomedicine strategy for preventing high-risk corneal transplantation rejection

Tuo Meng [a,1], Jinhua Zheng [a,b,1], Crystal S Shin [c], Nan Gao [d], Divya Bande [a], Hadi Sudarjat [a], Woon Chow [f,g], Matthew Sean Halquist [a], Fu-Shin Yu [d], Ghanashyam Acharya [c,e], Qingguo Xu [a,f,h] *

[a] Department of Pharmaceutics, Virginia Commonwealth University, Richmond, VA 23298, USA
[b] Department of Ophthalmology, Affiliated Hospital of Guizhou Medical University, Guiyang, Guizhou 550004, China
[c] Michale E. DeBakey Department of Surgery, Baylor College of Medicine, Houston, TX 77030, USA
[d] Departments of Ophthalmology, Anatomy and Cell Biology, Wayne State University School of Medicine, Detroit, MI 48201, USA
[e] Department of Materials Science and Nanoengineering, Rice University, Houston, TX, 77005, USA
[f] Department of Ophthalmology, Virginia Commonwealth University, Richmond, VA 23298, USA
[g] Department of Pathology, Virginia Commonwealth University, Richmond, VA 23298, USA
[h] Center for Pharmaceutical Engineering; Institute for Structural Biology, Drug Discovery & Development (ISB3D); and Massey Cancer Center, Virginia Commonwealth University, Richmond, VA 23298, USA

Supplementary methods:

Characterization of DSP-NP: A Zetasizer Nano ZS90 (Malvern Instruments, Southborough, MA) was used to determine the particle size, size distribution, and surface charge of DSP-NP. Transmission electron microscope (TEM) images of DSP-NP were obtained using a Hitachi H7600 transmission electron microscope (Hitachi Co. Ltd., Tokyo, Japan). To measure drug loading, 50 µl DSP-NP was lyophilized, weighed, and dissolved in 500 µl acetonitrile. 1 ml 50 mM EDTA was added to solubilize DSP-zinc complex, and the DSP concentration was measured by HPLC/UV analysis.

HPLC analysis was performed on a Shimadzu Prominence LC system (Kyoto, Japan) equipped with a Pursuit 5 C18 column (Varian Inc, Lake Forest, CA). Isocratic separation was conducted using the mobile phases consisting of acetonitrile/water (35/65 v/v) containing 0.1% trifluoroacetic acid (flow rate = 1 ml/min). Column effluent was monitored by UV detection at 241 nm.

Zinc and phosphorus quantification: Zinc and phosphorus were quantified using Inductively coupled plasma atomic emission spectroscopy (ICP-OES 5110; Agilent). Freeze dried DSP-NPs were dissolved in acetonitrile solution. Then the nanoparticle solution was further digested using 4% HNO_3 solution. Zinc and phosphorus standards were prepared by dissolving Zinc acetate and DSP in cell culture water, respectively. Then the standards were diluted with 4% HNO_3 solution to reach the range of 0~25 μg/ml. 2ml samples were injected for analysis and measurement was carried out using both Axial and Radial viewing mode conditions. Intensities (nm c/s) were recorded for every measurement.

Powder X-ray diffraction (PXRD): PXRD analysis was performed using Rigaku Miniflex 6G equipped with a D/tex Ultra scintillation detector (Rigaku, Japan). Freeze-dried Samples were exposed to Cu-Kα radiation source at 40 kV and 15mA, and scanned in the range of 3-50 °2θ with a 0.05 °2θ step width. The D/tex Ultra scintillation detector was used.

FTIR analysis of DSP and DSP-Zn complex: FTIR analysis was performed Nicolet iS50 FT-IR Spectrometer (Thermo Scientific, USA). DSP-Zn complex was prepared by adding 1 mL 0.5M Zn acetate solution to 0.5 mL DSP solution containing 10 mg DSP, and the complex was centrifuged, washed with DI water, and lyophilized ready for FTIR measurement.

DSC analysis of PLGA and PLA nanoparticles: The thermal properties of the $PLGA_{7kDa}$ and $PLA\text{-}2COOH_{8.2kDa}$ nanoparticle were analyzed using DSC (TA instrument, USA) with V4.5A thermal analysis Instruments. About 5 mg of the lyophilized nanoparticle were loaded and sealed in the aluminum pan. Samples were heated from 0 to 180°C at the heating rate of 10°C/min under the nitrogen environment. An empty pan was used as a reference.

Determination of carboxyl content in the polymers: Polymer carboxyl content was quantified by potentiometric titration [1-2]. Briefly, the polymer was dissolved in acetonitrile/water (80/20, v/v) at the concentration of 2.5 mg/ml. The polymer solution was titrated with 0.1M sodium hydroxide dropwise until the pH of the system reached the plateau. The pH of the solution was monitored using Fisher Scientific pH meter (MA, USA). The carboxyl content is expressed as μmol acid per gram polymer. Three replicates were performed for each polymer, and the results were expressed as mean ± SEM. The volume of base added was less than 9% of the total volume of the polymer solution to avoid the changing of the polymer pKa and the precipitation of the polymer.

ITC titration: ITC was performed using MicroCal PEAQ-ITC (Malvern Instruments, Southborough, MA). DSP-Zinc and $PLGA_{7kDa}$ was dissolved in anhydrous DMSO, yield the final concentration of DSP and $PLGA_{7kDa}$ carboxyl group at 7.5mM and 2mM, respectively. The standard set up included a total of 19 aliquots of DSP-Zn solution in DMSO titrated in 2 μl intervals from the syringe rotating at 750 rpm into the reaction

cell containing $PLGA_{7kDa}$ DMSO solution at 25°C. The captured heat change was corrected with the dilution heat change generated by injecting DSP-Zn solution into DMSO solution. The injection heat changes per mole of DSP-Zn were plotted against the molar ratio of the DSP to terminal free carboxyl groups in PLGA, fitted with a one-site independent model and analyzed with a nonlinear least squares regression algorithm by MicroCal PEAQ-ITC (Malvern Instruments, Southborough, MA).

Formulation of sunitinib malate loaded PLGA microparticles: 15 mg sunitinib malate dissolved in 200 µl DMSO and 75 mg $PLGA_{7kDa}$ was dissolved in 550 µl DCM, respectively. Then mixed the two solutions, and homogenized in 40 ml 1% PVA solution with the speed of 5000 rpm (IKA homogenizer) for 2 min. After that, the emulsion was poured to another 60 ml 0.3% PVA solution allowing for the evaporation of the organic solution in the chemical hood under stirring for 1h. To further remove the DCM from the system, the solution was placed in vacuum chamber for another 2~3 hours to remove the remaining organic solvent. Then the suspension was filtered through 40 µm strainer to remove large particles and washed with DI water for 4 times after centrifuged at 500g for 10 min. The SEM imaging of Sub MP was performed on Hitachi high-resolution analytical FE-SEM SU-70 (Hitachi Co. Ltd., Tokyo, Japan). Particle size and distribution of Sub MP was characterized using Malvern Mastersizer 3000 (Malvern Instruments, Southborough, MA). To measure drug loading, 50 µl Sub MP was lyophilized, weighed, and dissolved in DMSP. Sunitinib malate concentration was measured by UV–Vis at 441 nm on a BioTek Microplate Reader (Winooski, VT), and calculated using a standard curve of sunitinib malate in DMSO.

LC/MS/MS analysis of Axi in ocular tissues and plasma: Ocular tissues were directly homogenized in 250 µl of 90% acetonitrile-water solution containing 50 ng/ml internal standard (IS) Axi-d3 (Cat No: A794652, Toronto Research Chemicals, Toronto, Canada) using Next Advance Bullet Blender (Laboratory Supply Network, NH, USA). 50 µl of plasma/BSA standards were extracted with 200 µl of cold acetonitrile containing 50 ng/ml of the IS. After that, samples were centrifuged at 12000 g for 15 min and then the supernatant was collected for LC/MS/MS analysis. All ocular tissues were analyzed use 1x BSA standards and plasma samples were analyzed using plasma standards. Axi separation was achieved using Kinetex C18 column (2.1x100 mm, 2.6µm) at room temperature using a gradient with a flow rate of 0.38 mL/min. Mobile phase A was water containing 0.1% formic acid, and mobile phase B was acetonitrile containing 0.1% formic acid. The gradient started with 20% B holding for 1 min, then gradually increase to 95% B over 2 min. 95% B was hold for 1 min and then reduced to 20% B at 4.2 min. Finally, the column was equilibrated for 1.8 min. The whole process last around 6 min. The column eluent was monitored using Waters Xevo TQS micro Triple Quadrupole Mass Spectrometry system equipped with ESI source (Waters, MA, USA). Positive ionization mode was applied and the MRM transition of 387→356 (Axi) and 390→356 (Axi-d3) was monitored for Axi quantification. The limit of detection for Axi was 0.1 ng/ml.

Electroretinogram analysis (ERG): Rats were dark-adapted overnight, anesthetized by intramuscular injection of ketamine/xylazine, and placed on a heating pad set to 37 °C. The pupils were dilated with 1% tropicamide (Akron Pharmaceuticals, IL, USA) followed by 2.5% phenylephrine hydrochloride (Paragon BioTeck, OR, USA). A drop of 0.5% tetracaine hydrochloride eyedrop (Oceanside Pharmaceuticals, NJ, USA) was administered. After that, the 2.5% Hypromellose ophthalmic demulcent solution (Akron Pharmaceuticals, IL, USA) was applied followed by placing electrodes over the corneas. ERG responses were recorded by Espion ERG Diagnosys (MA, USA). Three intensities of lights (0.01, 0.1 and 1 cd.s/m^2) were tested for scotopic ERG first. Then two intensities of lights (3 and 10 cd.s/m^2) were measured for photopic ERG after a 10 min light adaptation. The a-wave and b-wave amplitudes were extracted using the Diagnosis Epsion software and transferred to Prism for analysis.

Intraocular pressure measurement: Non-invasive intraocular pressure (IOP) measurements were conducted using an Icare® Tonolab (Icare, Helsinki, Finland). The IOP of both eyes were measured. IOP value for each eye was from the average of three measurements and each measurement was from six consecutive readings.

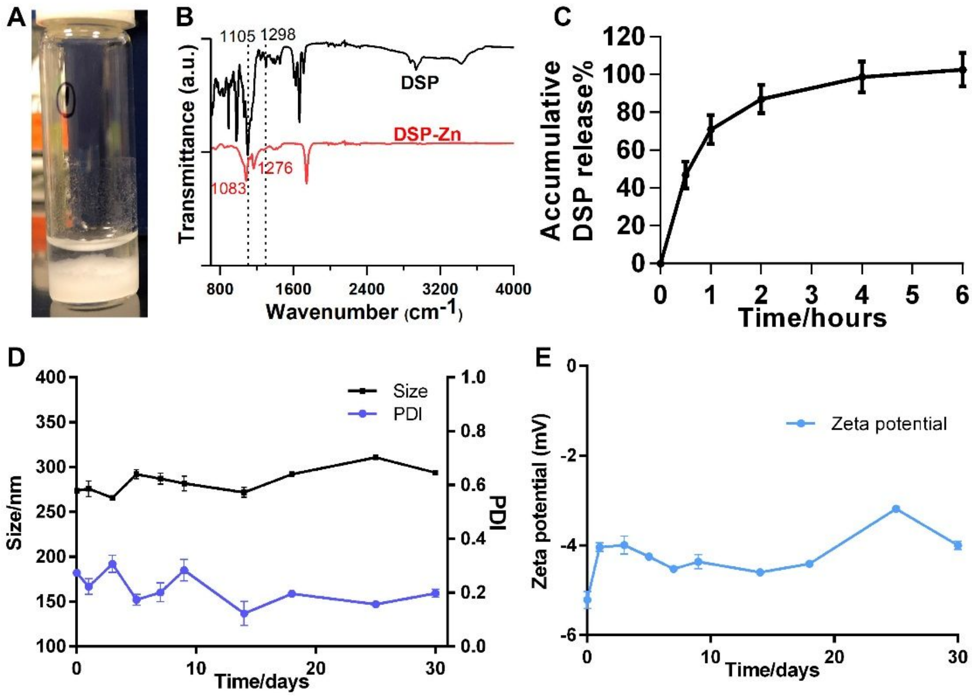

Fig S1: Formation and characterization of DSP-Zn complex **(A~C)**; colloidal stability of PLA DSP-NP under 5 ° C **(D~E)**. **(A)** Formation of DSP-Zn complex in water. **(B)** FTIR characterization of DSP-Zn complex. **(C)** In vitro DSP release profile of DSP-Zn. **(D)** PLA DSP-NP size, PDI and **(E)** zeta potential had no significant changes under 5°C storage over 30 days.

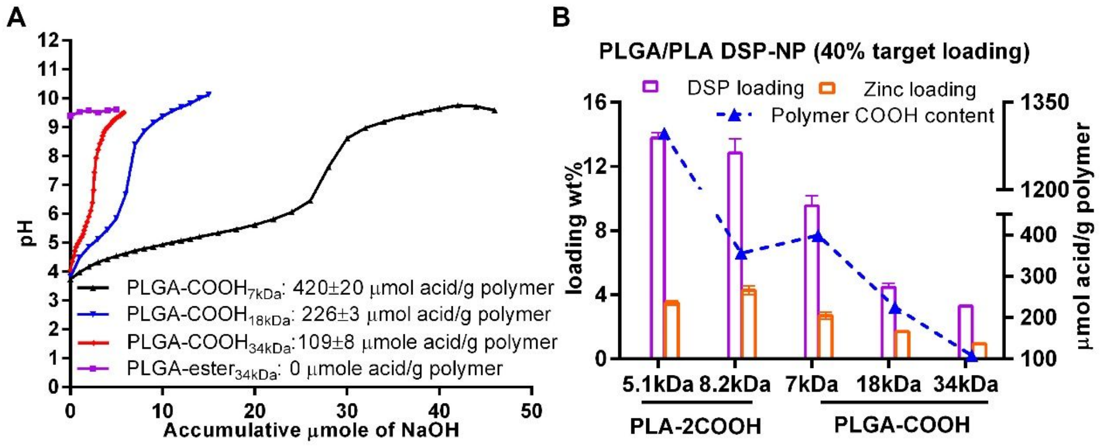

Fig S2: Correlation between carboxyl content in the PLGA polymer with the DSP/Zn loading in the nanoparticles. **(A)** Potentiometric titration of PLGA polymers. **(B)** DSP and Zn loading in the DSP-NP formulated with PLGA/PLA polymer with different carboxyl content. DSP-NP target drug loading is 40 wt% DSP. N=3 for each formulation.

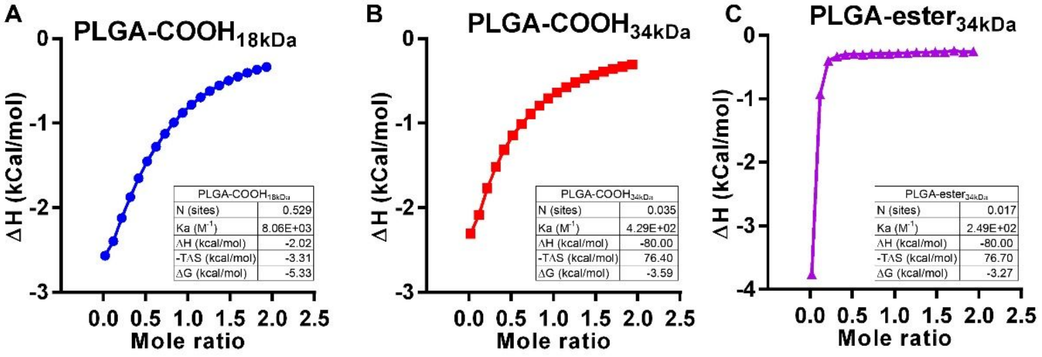

Fig S3: ITC titration cure of **(A)** PLGA-$COOH_{18kDa}$, **(B)** PLGA-$COOH_{34kDa}$ and **(C)** PLGA-$ester_{34kDa}$.

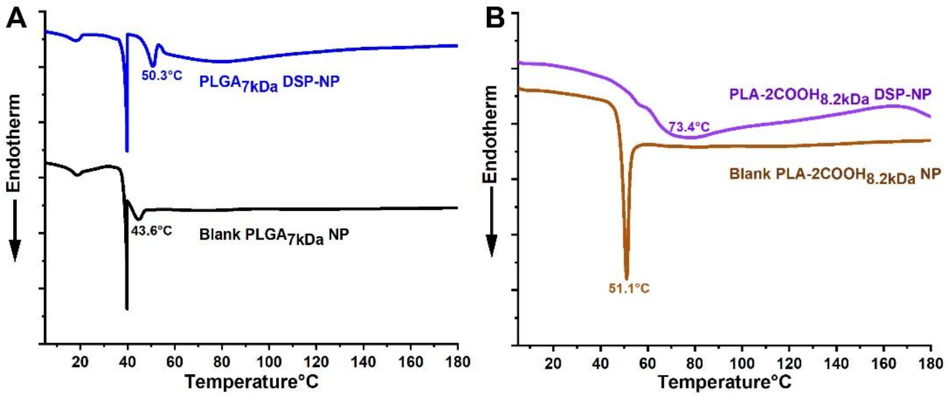

Fig S4: DSC analysis of **(A)** PLA-2COOH$_{8kDa}$, and **(B)** PLGA-COOH$_{7kDa}$ NPs.

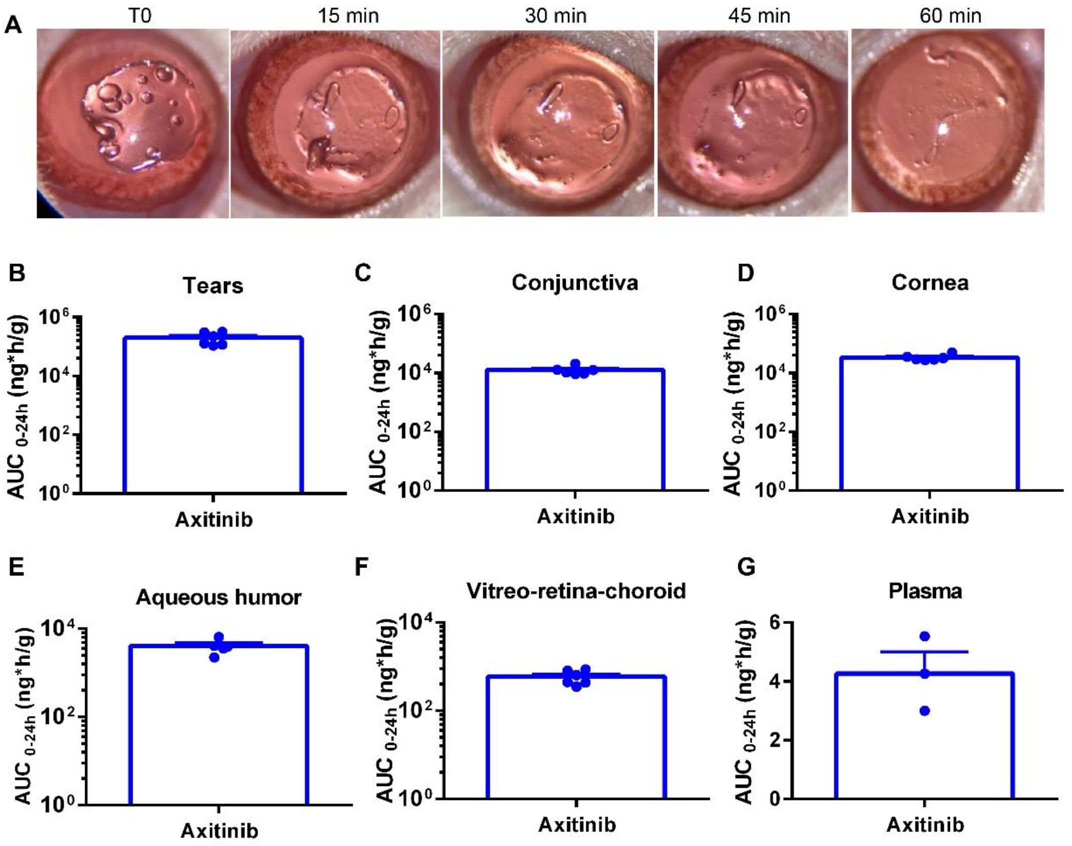

Fig. S5: NW dissolve process and AUC for Axi NW PK study. (A) NW gradually dissolved and disappeared within 60 min after placing on rat ocular surface. AUC for Axi ($AUC_{0\text{-}24h}$) after a single topical administration of Axi NW. **(B)** tears, **(C)** conjunctiva, **(D)** cornea, **(E)** aqueous humor, **(F)** vitreo-retina-choroid, and **(G)** plasma. AUC calculations were performed under non-compartment analysis using WinNonLin software. Data are presented as mean ± SEM (n = 3~6 for ocular tissues, n = 3 for plasma samples).

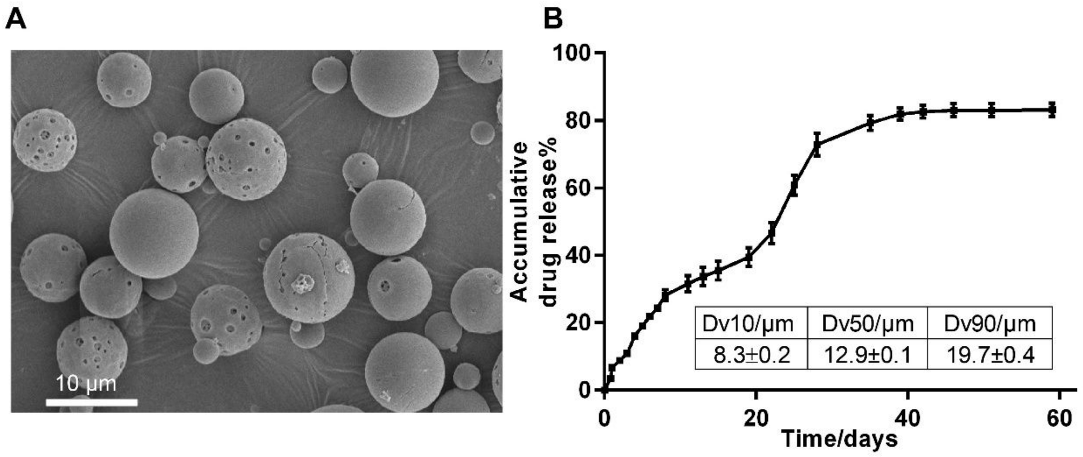

Fig S6: *In vitro* characterization of sunitinib malate loaded microparticle (Sub MP). Sunitinib malate loaded $PLGA_{7kDa}$ microparticle (Sub MP) was prepared using emulsification method as previously described [3]. Sunitinib malate was successfully encapsulated into PLGA microparticle with the drug loading of 9.6 wt%. **(A)** SEM analysis showed that Sub MP has a spherical shape with the pores presenting on the microparticle surface. **(B)** *In vitro* drug release analysis demonstrated that Sub-MP could provide sustained drug release up to 2 months. N=3 samples for *in vitro* drug release analysis.

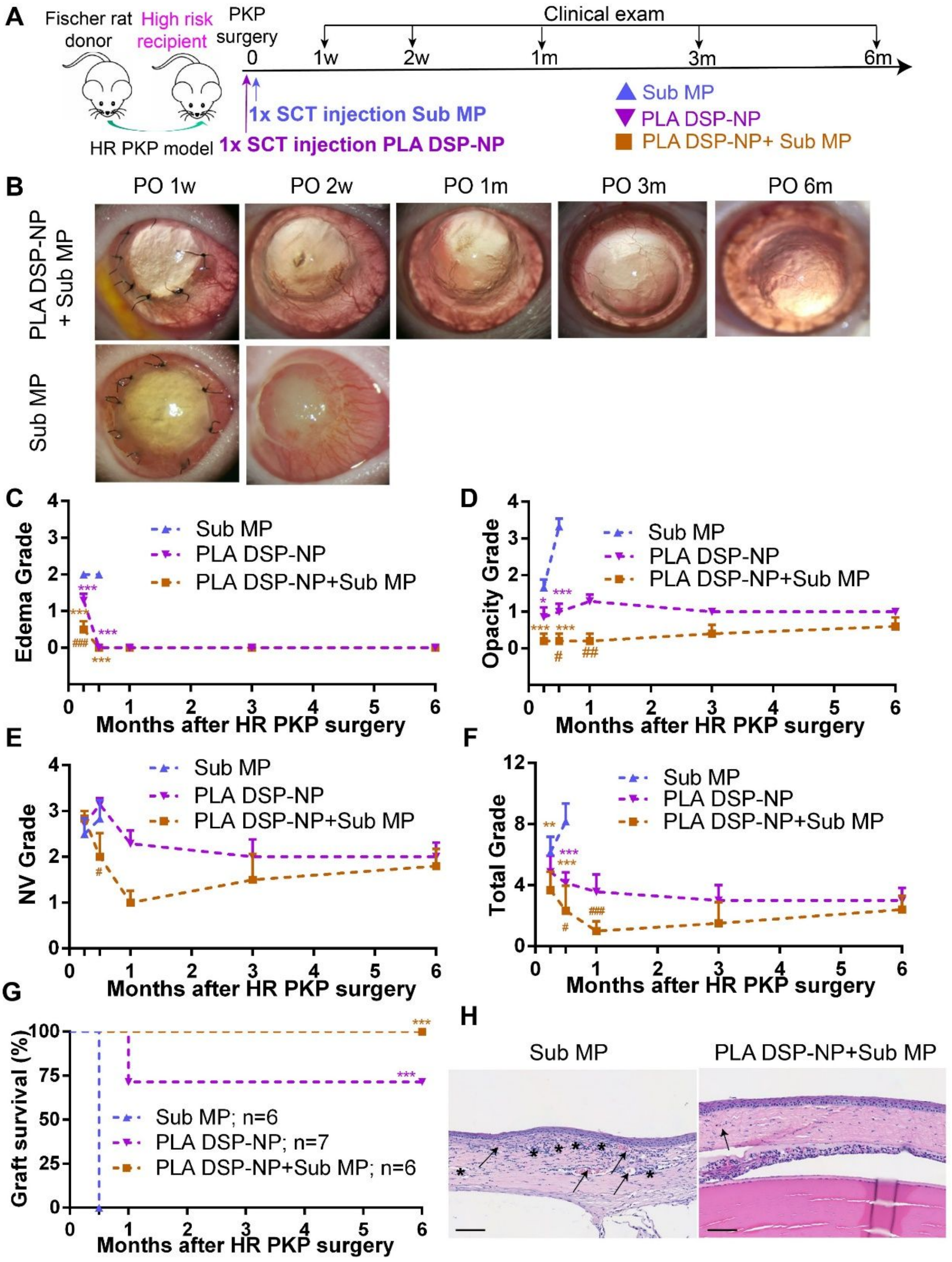

Fig S7: Efficacy of PLA DSP-NP and Sub MP in preventing high-risk corneal allograft rejection. (A) The schedule for the efficacy study of PLA DSP-NP and Sub MP in preventing high-risk corneal allograft rejection. **(B)** Representative grafts images at 1w, 2w, 1m, 3m and 6m after high-risk PKP (HR PKP) surgery. **(C)** Edema**, (D)** opacity, **(E)** neovascularization, **(F)** total clinical grade and **(G)** survival curves of grafts received different treatment after HR PKP surgery. Sub MP only treatment cannot prevent corneal grafts rejection and all grafts were rejected at PO 2w. Whereas, the PLA DSP-NP and Sub MP combination treatment provided 100% graft survival rate at

PO 6m and the overall corneal opacity grade <1. **(H)** Histology analysis showed that the rejected grafts received Sub MP only treatment had obvious inflammatory cell infiltration (black asterisk) together with a notable amount of NV detected in the cornea grafts (black arrow); While, the corneal grafts received combination treatment has no inflammatory cell infiltration nor edema, and only a limited amount of cornea NV (black arrow) was observed. Scale bar 100 μm. Statistical analysis for (C~G): two-way ANOVA followed by Bonferroni post hoc test for multiple comparison. (* indicates analysis versus Sub MP only treatment; ** $p \leq 0.01$; *** $p \leq 0.001$; # indicates analysis versus combination treatment; # $p \leq 0.05$, ## $p \leq 0.01$, ## $p \leq 0.001$). All data is plotted from mean ± SEM.

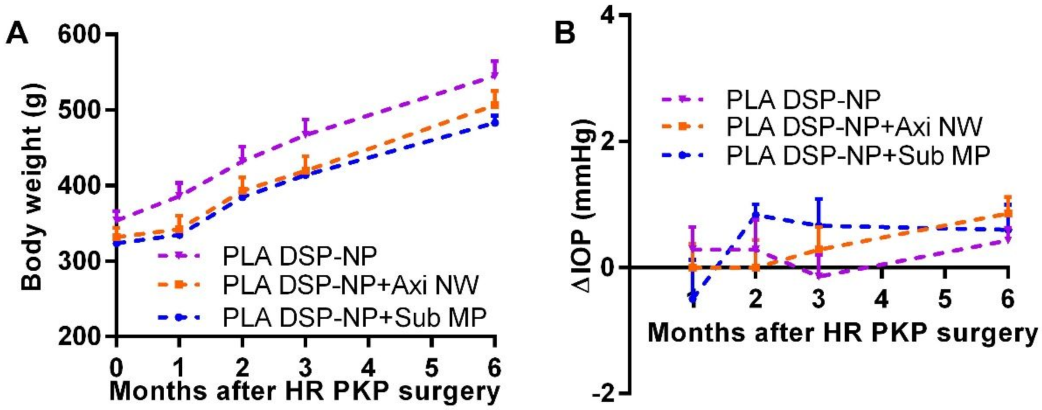

Fig. S8: Rats BW and IOP follow up after the high-risk PKP surgery. No obvious (A) BW reduction and (B) IOP increase was detected during the 6 months follow up after the high-risk PKP surgery. Data are presented as mean ± SEM (n = 5~7).

Table S1: Characterization of PLGA/PLA DSP-NPs.

Formulation	Target DSP loading wt%	Zinc/Phosphorus (mole ratio)	Particle size/nm	PDI
PLGA-$COOH_{7kDa}$	20	1.8	189±13.6	0.152±0.013
PLGA-$COOH_{18kDa}$		2.5	230±12.3	0.094±0.058
PLGA-$COOH_{34kDa}$		2.4	295±25.2	0.097±0.01
PLGA-$ester_{34kDa}$		NA	378±35.8	0.241±0.053
PLA-$2COOH_{5.1kDa}$		2.2	187±64.5	0.173±0.055
PLA-$2COOH_{8.2kDa}$		2.3	267±65.0	0.185±0.017
PLGA-$COOH_{7kDa}$	40	2.1	190±12.2	0.192±0.03
PLGA-$COOH_{18kDa}$		2.5	253±26.3	0.073±0.05
PLGA-$COOH_{34kDa}$		1.7	283±17.9	0.214±0.019
PLA-$2COOH_{5.1kDa}$		2	284±18.6	0.148±0.052
PLA-$2COOH_{8.2kDa}$		2.2	331±16.6	0.267±0.048

Table S2: Primer sequences used in RT-PCR analysis.

Gene	Forward primer (5'~3')	Reverse primer (5'~3')
LYVE-1	GCCAAGGAGGTCTGTAAGGT	AACCCATCCATAGCTGCAAG
CD31	CAGCCATTACGACTCCCAGAA	AGCCTTCCGTTCTCTTGGTG
VEGFA	GGATCAAACCTCACCAAAGCC	TTGTTCTATCTTTCTTTGGTCTGC
VEGFC	GTTACCTCAGCAAGACGTTGT	CACCGGCAGGAAGTGTGATT
VEGF-R1	GGAAGACTCGGGCACCTATG	AGTGGCGCTTCCAAATCTCT
VEGFR2	ATTCCCGTCCTCAAAGCATCA	ACGCTGTGCAGGTGTATTCT
VEGFR3	GAACTTGACCGACCTCCTGG	CCAGGTCGATTCCCGACTCT

Table S3: Clinical evaluation of HR recipients after corneal transplantation (score: 0~4) [4].

Score	Transparency	Neovascularization	Edema
0	Clear	None	None
1	Slightly opacity	Neovascularization invading to 1/3 of recipient	Slight
2	Mild opacity with iris details visible	Neovascularization invading to 2/3 of recipient	Moderate stromal edema
3	Moderate opacity, iris details not visible	Neovascularization invading to the edge of graft	Marked stromal edema
4	Serve opacity, white cornea	Neovascularization invading to the graft	Serve edema

References:

1. Beig, A.; Feng, L.; Walker, J.; Ackermann, R.; Hong, J. K. Y.; Li, T.; Wang, Y.; Qin, B.; Schwendeman, S. P., Physical-Chemical Characterization of Octreotide Encapsulated in Commercial Glucose-Star PLGA Microspheres. Mol. Pharmaceutics **2020,** *17* (11), 4141-4151.
2. Hong, J. K. Y.; Schwendeman, S. P., Characterization of Octreotide–PLGA Binding by Isothermal Titration Calorimetry. **2020,** *21* (10), 4087-4093.
3. Yang, J.; Luo, L. X.; Oh, Y. M.; Meng, T.; Chai, G. H.; Xia, S. Y.; Emmert, D.; Wang, B.; Eberhart, C. G.; Lee, S.; Stark, W. J.; Ensign, L. M.; Hanes, J.; Xu, Q. G., Sunitinib malate-loaded biodegradable microspheres for the prevention of corneal neovascularization in rats. J Control Release **2020,** *327*, 456-466.
4. Pan, Q.; Xu, Q.; Boylan, N. J.; Lamb, N. W.; Emmert, D. G.; Yang, J. C.; Tang, L.; Heflin, T.; Alwadani, S.; Eberhart, C. G.; Stark, W. J.; Hanes, J., Corticosteroid-loaded biodegradable nanoparticles for prevention of corneal allograft rejection in rats. J Control Release **2015,** *201*, 32-40.
